# Genetic and molecular factors associated with changes in structural-functional coupling in medication-free obsessive-compulsive disorder

**DOI:** 10.1017/S0033291726103389

**Published:** 2026-02-20

**Authors:** Xiaolu Zhang, Na Liu, Xuedi Zhang, Jieling Xu, Minyao Xie, Chenchen Shao, Yue Sun, Yuxin Li

**Affiliations:** 1 Nanjing Medical University Affiliated Brain Hospital: Nanjing Brain Hospital, Nanjing, China; 2 Department of Medical Psychology, Nanjing Medical University Affiliated Brain Hospital: Nanjing Brain Hospital, China; 3 Nanjing Normal University, China

**Keywords:** Allen Human Brain Atlas, gene expression, imaging genetics, neuroimaging, obsessive-compulsive disorder, structure–function coupling

## Abstract

Obsessive-compulsive disorder (OCD) is a complex psychiatric disorder. While existing studies have revealed abnormalities in brain structure and function associated with OCD, there is a paucity of research integrating these two aspects, and the transcriptional patterns underlying these abnormalities remain unclear. This study is a multiscale, exploratory investigation designed to generate hypotheses rather than to test causal mechanisms. We aimed to investigate aberrations in brain structure–function coupling (SFC) in OCD patients and, by integrating gene expression profiles and neurotransmitter maps, to explore the potential molecular and genetic bases of these changes. We recruited 100 medication-free OCD patients and 90 healthy controls, and employed multimodal imaging techniques to systematically analyze abnormalities in static SFC in OCD patients. Subsequently, we conducted transcriptomic analysis to identify genes associated with SFC abnormalities and performed spatial correlation analysis with neurotransmitter atlases to investigate potential links between SFC dysregulation and transcriptional patterns. Our findings demonstrated that OCD patients exhibit significant SFC abnormalities in the right temporoparietal junction (rTPJ). These SFC abnormalities are significantly associated with 2,421 gene expression profiles and the serotonin neurotransmitter system. Gene enrichment analysis revealed that these aberrant genes are primarily involved in key biological processes, such as brain development, synaptic signaling, cell projection development, and regulation of neuronal processes. By integrating multimodal imaging, transcriptomic, and neurotransmitter data, this study provides multiscale evidence for the potential molecular basis of SFC abnormalities in the rTPJ of OCD patients, offering preliminary insights into a possible pathological pathway of OCD.

## Introduction

Obsessive-compulsive disorder (OCD) is a chronic psychiatric disorder, typically characterized by fluctuating symptoms, as well as obsessions and compulsions. The lifetime prevalence rate of OCD is between 1% and 3% (Huang et al., 2019; Rao et al., 2018). Neuroimaging studies have frequently focused on structural and functional abnormalities in the cortico-striato-thalamo-cortical (CSTC) neural circuitry (Rao et al., 2018; Simon, Adler, Kaufmann, & Kathmann, 2014; H. Zhang et al., 2019). Moreover, other brain regions (such as the frontoparietal lobe, temporal lobe, and cerebellum) have also been found to be involved in the pathological processes of OCD (Fan et al., 2017; Qiu et al., 2017; Zhao et al., 2017). Therefore, understanding the complex mechanisms of OCD requires moving beyond a single-circuit perspective.

In terms of brain structure, studies have shown that patients with OCD exhibit a significant reduction in fractional anisotropy of white-matter microstructures, including the corpus callosum (a key fiber tract connecting the prefrontal lobes). This reduction leads to abnormal communication between the prefrontal lobes, thereby impairing cognitive flexibility (Radua et al., 2014). Regarding brain function, OCD patients also display characteristic connectivity abnormalities, including enhanced connectivity in the caudate-frontal limbic network and diminished connectivity in the caudate-frontal-parietal network (J. Liu et al., 2022). With the advancement of research, it is now recognized that differences in brain structure and function are not independent; their interdependence has gradually gained acceptance, and the cerebral cortex presents a hierarchical and ordered organizational pattern in both structure and function. To better convey these findings, researchers use the concept of structure–function coupling (SFC) (Fotiadis et al., 2024). Research on SFC holds significant value for unraveling the mechanisms underlying functional integration among different brain regions, particularly in elucidating the pathological mechanisms of diseases (Luo et al., 2020; Preti & Van De Ville, 2019). In depression (Liao et al., 2024) and schizophrenia (Sun et al., 2017), weakened coupling in the frontal-subcortical pathways and decoupling of limbic system modules have been observed, which lead to abnormalities in executive function and emotional regulation, suggesting that SFC could serve as a transdiagnostic biomarker. In the field of OCD, existing studies have only identified a potential specific imbalance pattern between the decoupling of SFC in the goal-directed network and SFC in the habit network (Xu et al., 2022). While this provides a new perspective on the pathological mechanisms of OCD, it urgently requires validation due to sample limitations (only 30 male patients). This study incorporates the SFC perspective, aiming to offer unique mechanistic insights into OCD and thereby enhance the diagnostic accuracy of the disorder.

The pathological mechanisms of OCD involve interactions between genetic susceptibility and neurotransmitter systems. Genetic studies have confirmed that OCD exhibits a significant heritability (Zai et al., 2019), with specific genetic variations capable of influencing brain imaging phenotypes (e.g. serotonin transporter function and white matter structure) (Atmaca et al., 2010; Hesse et al., 2011). Large-scale genetic analyses (such as Genome-Wide Association Studies [GWAS]) have further identified OCD risk loci (Strom et al., 2024). Critically, genetic risks may affect the structure–function integration of specific brain circuitries by altering the expression of neurotransmitter-related genes (e.g. serotonin, dopamine, and glutamate pathways) (Hesse et al., 2011; Li et al., 2024; Pauls, Abramovitch, Rauch, & Geller, 2014). This gene-transmitter-circuitry cascade effect provides a theoretical framework for exploring the molecular basis of imaging abnormalities in OCD in the present study.

Imaging genetics integrates the fields of neuroimaging and molecular genetics to explore how genes conferring susceptibility act on the brain. This integration potentially offers a more precise understanding of the pathophysiological mechanisms underlying OCD. The Allen Human Brain Atlas (AHBA), which is derived from post-mortem tissue samples of six healthy adult donors, quantifies the expression levels of more than 20,000 genes (M. J. Hawrylycz et al., 2012; M. Hawrylycz et al., 2015; Sunkin et al., 2013). By combining neuroimaging data with gene transcription information from the Allen Atlas, researchers discovered that gray matter morphological abnormalities in OCD are significantly correlated with dopamine synthesis capacity and the expression profiles of 1,110 genes (Li et al., 2024). Another study has also identified over 1,000 differentially expressed genes associated with OCD-related cortical changes (D. Zhang et al., 2024). These studies, based on the AHBA, have explored the transcriptional and molecular underpinnings of neuroimaging phenotypes in OCD, thereby providing new insights into a deeper understanding of the disorder.

While neuroimaging studies have separately revealed abnormalities in brain structure and function in OCD (J. Liu et al., 2022; Radua et al., 2014), there is a paucity of research linking these two aspects. The transcriptional mechanisms underlying such abnormalities and their associations with clinical features remain unclear. By integrating multimodal imaging, transcriptomic, and neurotransmitter mapping data, this study aims to conduct exploratory research with the following objectives: (1) Exploratively identify abnormalities in resting-state SFC in medication-free patients with OCD; (2) Explore the relationships between these abnormalities and the clinical features of OCD; (3) Elucidate the multiscale mechanisms of brain regions with data-driven SFC abnormalities: on the one hand, analyzing and associating their gene expression profiles via the AHBA, and on the other hand, evaluating their neurochemical association patterns based on neurotransmitter maps. The innovation of this study lies in taking SFC as the core integration node, integrating data from the AHBA and neurotransmitter maps to achieve synergistic analysis of multimodal information. It seeks to outline a multiscale framework that links transcriptional patterns, SFC, and clinical features, suggesting a possible cross-scale interpretation for OCD pathology.

## Methods and materials

### Participants

We recruited 100 OCD patients who met the Diagnostic and Statistical Manual of Mental Disorders, Fifth Edition diagnostic criteria and had not received any current or recent (≥8 weeks) pharmacological treatment from the outpatient and inpatient departments of the Department of Medical Psychology at the Affiliated Brain Hospital of Nanjing Medical University. In addition, 90 healthy controls (HCs), matched for gender and age, were recruited via local advertisements. All participants gave their written consent, and the study received approval from the Ethics Committee of the Affiliated Brain Hospital of Nanjing Medical University (IRB NO. 2020KY208-01). The Supplementary Materials contain the inclusion and exclusion criteria.

### Clinical assessment

All participants provided self-reported information on their ethnicity and handedness. They were also assessed using the Yale–Brown obsessive compulsive scale (YBOCS) (Storch et al., 2010), the Obsessive-Compulsive Inventory-Revised (OCI-R) (Wootton et al., 2015), the Beck Anxiety Inventory (BAI) (Beck, Epstein, Brown, & Steer, 1988), the Beck Depression Inventory-Second Edition (BDI-II) (Wang & Gorenstein, 2013), and the Sheehan Disability Scale (SDS) (Abdin et al., 2024). The Mini International Neuropsychiatric Interview was used to assess the comorbid conditions (Sheehan et al., 1998). Notably, no enrolled participants met the criteria for a current substance use disorder. Among the 100 medication-free patients with OCD, 12 had comorbid Generalized Anxiety Disorder, and 8 were currently experiencing comorbid major depressive episode.

### Brain imaging data

Whole-brain resting-state Magnetic Resonance Imaging (MRI) images were acquired using a Siemens 3.0T scanner. This imaging was conducted in the Department of Radiology at the Affiliated Brain Hospital of Nanjing Medical University. This process was performed using the DPARSF V3.0 toolkit of the SPM8 toolkit (Yan, Wang, Zuo, & Zang, 2016). After removing the maximum head displacement of >3 mm and the maximum rotation head motion of more than 3°, there were 84 HCs and 96 OCDs. Brain structural data were processed based on DSI Studio (http://dsi-studio.labsolver.org) (Tax et al., 2022; Yeh et al., 2013). Detailed information on magnetic resonance imaging (MRI) data acquisition parameters and image preprocessing procedures is shown in Supplementary Data 1.

### Construction of structural-functional coupling

The whole-brain time series was extracted from the preprocessed functional MRI (fMRI) data, and the correlation coefficient was calculated using Pearson’s correlation. The correlation *z*-value was converted to the Fisher’s coefficient, and the functional network connection matrix was constructed using the Schaefer400 atlas (Schaefer et al., 2018). The structural imaging data were matched to the Schaefer400 atlas through whole-brain fiber tracking, and the number of fibers was calculated to obtain the fiber number matrix. Then, the structural-functional coupling is constructed through Pearson correlation. The detailed steps can be found in the Supplementary Materials. We conducted intra-module and inter-module connection coupling level analysis, and age, education level, Framewise Displacement (FD), and handedness were regressed as potential confounding factors in the analysis. The Bonferroni correction approach was implemented, and the threshold for statistical significance was defined as *p* < 0.05, subsequent to Bonferroni correction. In addition, we reanalyzed the key brain regions using the Human Connectome Project Multimodal Parcellation (HCP-MMP) atlas.

### Validation analysis: calculation of topological properties of brain functional networks

Statistical parameter mapping (SPM12, https://www.fil.ion.ucl.ac.uk/spm/) and the GRETNA package in the MATILAB 2021b toolbox were used to calculate the network properties of the functional network, including small-world properties, clustering coefficient, shortest path length, global efficiency, local efficiency, and node parameters, including degree centrality and node efficiency, to examine the network topological characteristics of the entire brain. Age, educational attainment, FD, and handedness were regressed as potential confounding factors in the analysis. Additionally, the Bonferroni correction method was employed, and the statistical significance was set at *p* < 0.05 (after Bonferroni correction). At the same time, the node properties were compared with the results of the structural-functional coupling brain regions as a validation.

### Clinical significance of functional connectivity changes

In order to determine the clinical significance of abnormal changes in brain structural functional coupling, we performed Pearson’s correlation analysis on the coupling changes and clinical measurements (YBOCS, OCIR, BAI, and BDI-II scores). To evaluate the relationship between alterations in SFC and clinical manifestations.

### Abnormal structural function coupling and gene expression

Gene expression data were obtained from the AHBA (http://human.brain-map.org), comprising left hemisphere samples from six adult donors (due to limited right hemisphere availability). Data were preprocessed using the abagen toolbox with default parameters and mapped to the Schaefer400 atlas. We applied differential stability scores to identify consistent gene expression patterns, retaining only the top 50% of genes based on inter-donor Spearman correlations of regional expression profiles (Arnatkevic, 2019).

Partial least squares (PLS) regression was employed to examine associations between gene expression and OCD-related SFC differences. Gene weights were evaluated through 5,000 bootstrap iterations, with significant genes identified using *z*-scores (weight/bootstrap standard error) (Bigdeli et al., 2016). This approach generated our final list of OCD-relevant genes while controlling for multiple comparisons.

### Gene enrichment analysis

Metascape (https://metascape.org) (Zhou et al., 2019) was used to perform Gene Ontology (GO) analysis and Kyoto Encyclopedia of Genomes (KEGG) pathway analysis. These analyses were conducted on the identified genes. Metascape uses the hypergeometric test and the Benjamin–Hochberg *p*-value correction algorithm to identify genes with a significantly larger number of genes included in the ontology terms. GO was used to identify biological functions, including molecular functions, biological processes, and cellular components (Thomas et al., 2022). The KEGG helped identify related biological pathways (Kanehisa et al., 2017).

### Spatial correlation between abnormal SFC and neurotransmitter systems

In this study, we investigated the spatial correlation between abnormal SFC and receptor/transporter density. Specifically, we calculated the spatial correlation between abnormal SFC (non-thresholded *t*-statistics) and neurotransmitter maps (including 5-HT, DA, Glu, etc.) provided by the JuSpace toolbox (Dukart et al., 2018, 2021). Correlation analyses were performed using the Schaefer400 brain atlas, with statistical evaluation conducted via 5,000 permutation tests. All results were corrected using the False Discovery Rate (FDR) method to ensure their reliability. For further details, please refer to the Supplementary Materials.

## Results

### Clinical and demographic characteristics

The demographic data of all participants are shown in Table S1 of Supplementary Data 1. There was no statistical difference in age, gender, and ethnicity between the OCD and HC groups (*P* > 0.05). The years of education in the OCD group were significantly lower than those in the HC group (*P* < 0.05). In terms of clinical characteristics, the YBOCS, BAI, BDI-II, OCI-R, and SDS scores of HC and OCD patients were significantly different (*p* < 0.05).

### Imaging results

#### Structural-functional coupling

At the whole-brain level, as well as within and between the seven networks, no significant differences in structure–function connectivity coupling were observed between the OCD group and the HC group (whole-brain level, *t* = −1.439, *p* = 0.152). The results of the coupling between the seven networks are provided in Table S4 of Supplementary Data 1.

#### Brain region structural function coupling

**Figure 1.**
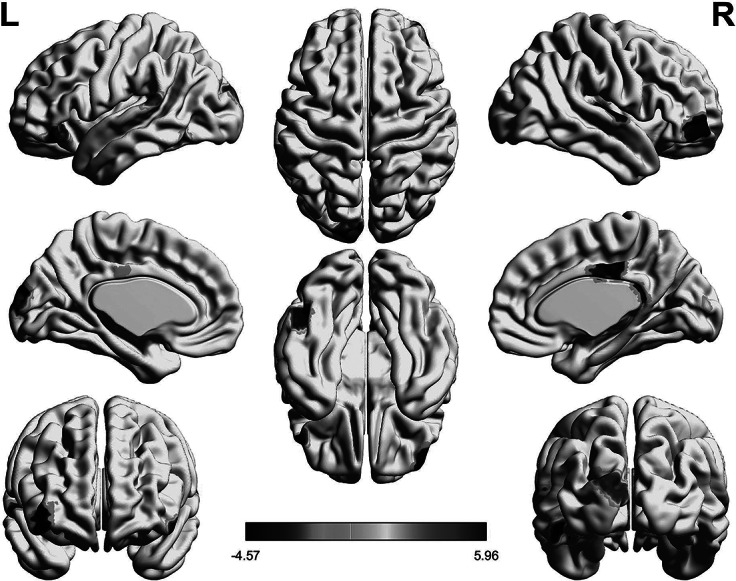
Results of brain region coupling: Compared with the HC group, the differences in the structural-functional coupling of brain regions in the OCD group.

In addition, 400 brain regions were coupled separately, and it was found that the two groups had significant differences in the structural function of the upper part of the lateral stripe in the left peripheral visual cortex, bilateral supplementary motor areas (SMAs), the left parietal lobe, the left temporal lobe, the right prefrontal cortex (PFC), and the right temporoparietal junction (rTPJ) (Figure 1 and Supplementary Table S5) (Xia, Wang, & He, 2013). In the HCP-MMP atlas, the regions corresponding to the rTPJ are R_TPOJ1 and R_TPOJ2. Both regions showed statistical trends consistent with the main analysis (R_TPOJ1: *t* = 2.5420, *p* = 0.0119; R_TPOJ2: *t* = 2.6295, *p* = 0.0093) (Supplementary Table S9). After Bonferroni correction, however, these findings did not remain statistically significant (*p*_corrected >0.05).

#### Changes in the topology of the whole-brain functional network

The results of the two independent sample *t*-tests showed that there was no difference between the two groups in small worldness, clustering coefficient, and local efficiency. However, in terms of global efficiency, the OCD group was significantly lower than the HC group (*t* = −2.120, *p* = 0.029), and in terms of the shortest path length, the OCD group was significantly higher than the HC group (*t* = 2.749, *p* = 0.034). In terms of degree centrality and node efficiency, the two groups showed significant differences in the rTPJ ([*t* = −4.2827, *p* = 0.0122) [*t* = −4.6866, *p* = 0.0022]). After comparison, it was found that rTPJ had differences in structural functional coupling and network topological properties (Tables S6 and S7).

### Pearson correlation analysis between structural-functional connectivity coupling and clinical scores

**Figure 2.**
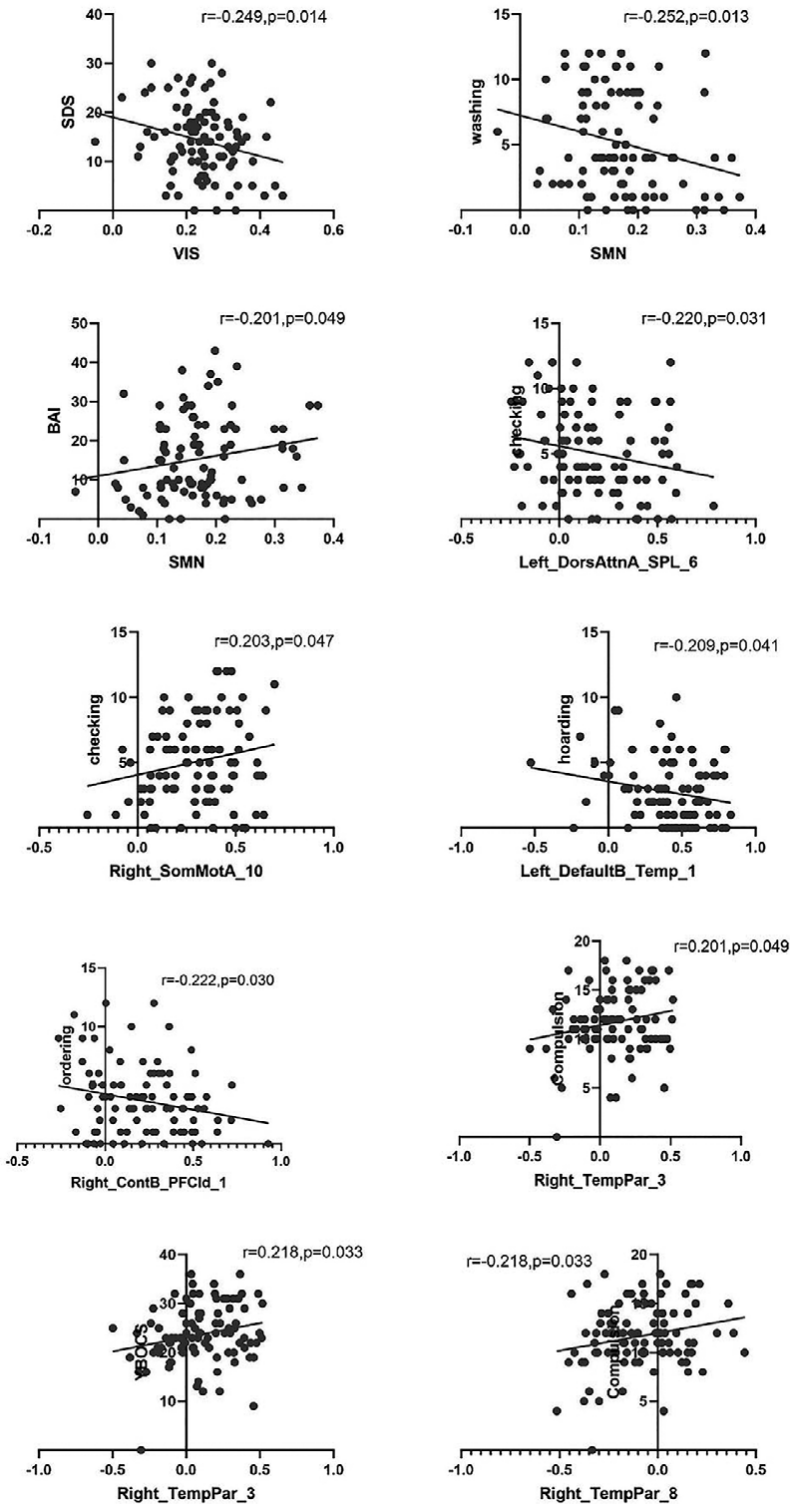
Pearson correlations between clinical characteristics and network topology indicators in all patients with OCD.

Pearson correlation analysis showed that the visual network was negatively correlated with the daily function of OCD (*r* = −0.249, *p*<0.05). The somatic motor network was significantly negatively correlated with the washing dimension of OCD patients and positively correlated with anxiety symptoms (*r* = −0.252, *r* = 0.201, *p*<0.05). In addition, the rTPJ was significantly positively correlated with the severity of OCD, especially compulsions (*r* = 0.218, *r* = 0.201, *p*<0.05). There is a negative correlation between the left parietal lobe and the inspection dimension (*r* = −220, *p*<0.05). There is a negative correlation between the left temporal lobe region and hoarding behavior (*r* = −0.209, *p*<0.05). The PFC is negatively correlated with ordering behavior (*r* = −0.222, *p*<0.05) (Figure 2).

### Change pattern of structural-functional coupling in OCD and its gene expression

**Figure 3.**
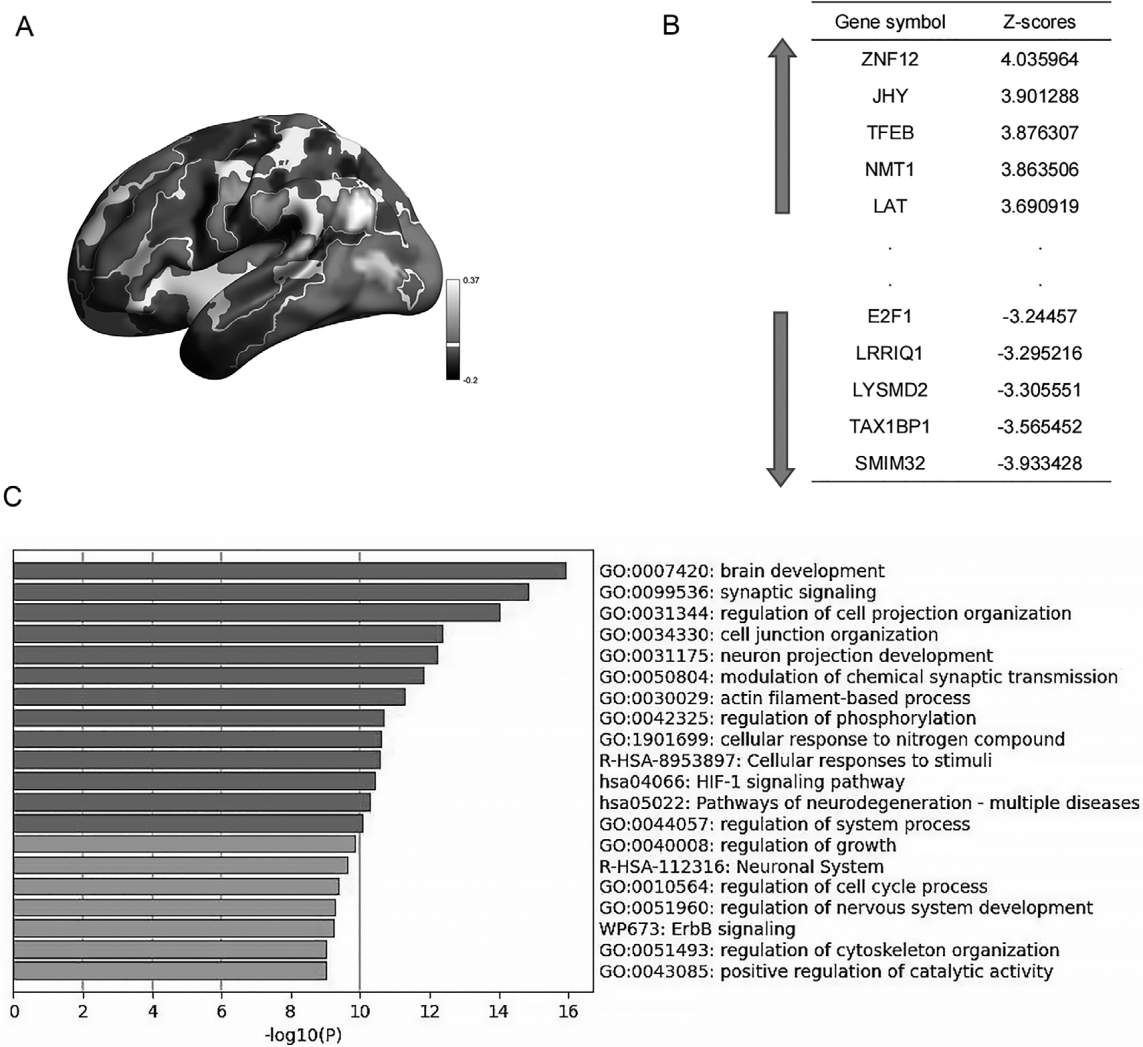
Results of genes associated with abnormal structural-functional coupling and their enrichment pathways, as shown in PLS. (a) The gene-weighted expression atlas (PLS1 weight Z-map) obtained by PLS regression analysis. (b) Gene list sorted by PLS1. PLS+ represents highly expressed genes with a positive-direction weight, and PLS- represents low expressed genes with a negative-direction weight. (c) The top 20 enrichment terms of genes.

To explore the potential molecular correlates, we performed a PLS regression analysis between the pattern of structural-functional coupling abnormality and the available whole-brain gene expression data from the AHBA. This exploratory analysis revealed that PLS1 (the first component of PLS regression) explained 21.81% of the variation in the structural-functional change pattern of OCD (permutation test, *P* < 0.05) (Figure 3a). Based on this model, we identified 2,421 genes (1,176 positive genes and 645 negative genes) were significantly associated with abnormal outcome functions in patients with OCD (Figure 3b). Subsequent GO and KEGG enrichment analysis was performed on the genes that contributed significantly in PLS1+ and PLS1- in the PLS1 gene list associated with abnormal OCD structural-functional coupling, and the most significant Top20 pathways after enrichment were reported (Figure 3c). According to the GO database, the significantly contributing genes were enriched in brain development, synaptic signaling, regulation of cell process organization, cell junction organization, neuronal process development, regulation of chemical synaptic transmission, actin filament-based processes, phosphorylation regulation, cell response to nitrogen compounds, cell response to stimulation, Hypoxia‑inducible factor 1 (HIF‑1) signaling pathway, pathways of neurodegeneration, and regulation of systemic processes (FDR correction *q* < 0.01).

### Relationship between abnormal SFC and neurotransmitter systems

**Figure 4.**
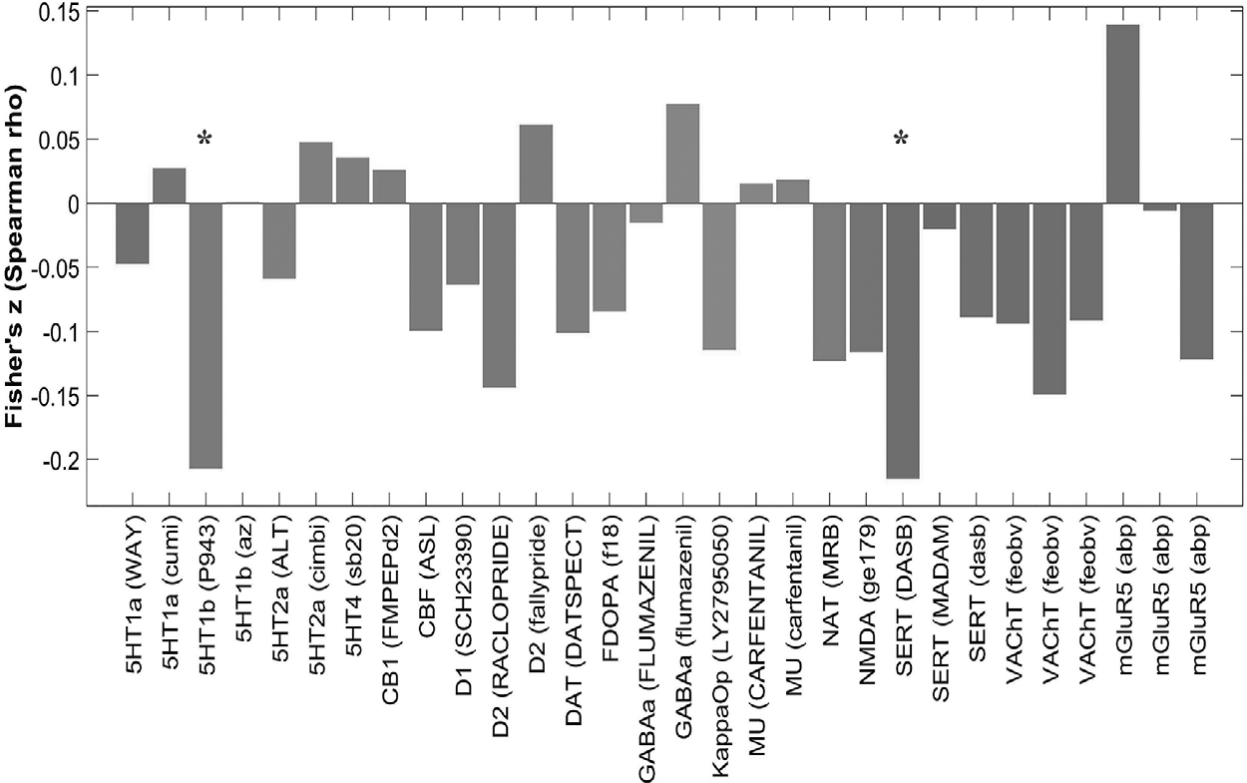
Association between structural-functional coupling abnormalities and neurotransmitter systems. The red star represents that the correlation is significant (FDR corrected *p* < 0.05). *Note*: 5-HT1a, 5-HT subtype 1a; 5-HT1b, 5-HT subtype 1b; 5-HT2a, 5-HT subtype 2a; D1, dopamine D1; D2, dopamine D2; DAT, dopamine transporter; F-DOPA, dopamine synthesis capacity; NAT, noradrenaline transporter; SERT, serotonin transporter; 5-HT4, 5-HT subtype 4; CB1, cannabinoid receptor 1; CBF_ASL_MRI, an MRI technique for measuring cerebral blood flow; GABAa, gamma-aminobutyric acid A receptor; KappaOp, kappa opioid receptor; MU, mu opioid receptor; NMDA, N-methyl-D-aspartate receptor; SERT, 5-hydroxytryptamine transporter; VAChT, vesicular acetylcholine transporter; mGluR5, metabotropic glutamate receptor 5.

Abnormal SFC was found to be significantly correlated with 5HT1b (Spearman’s *r* = −0.21, FDR corrected, *p* = 0.045), and with serotonin transporter (SERT) (Spearman’s *r* = −0.22, FDR corrected, *p* = 0.024) (Figure 4).

## Discussion

**Figure 5.**
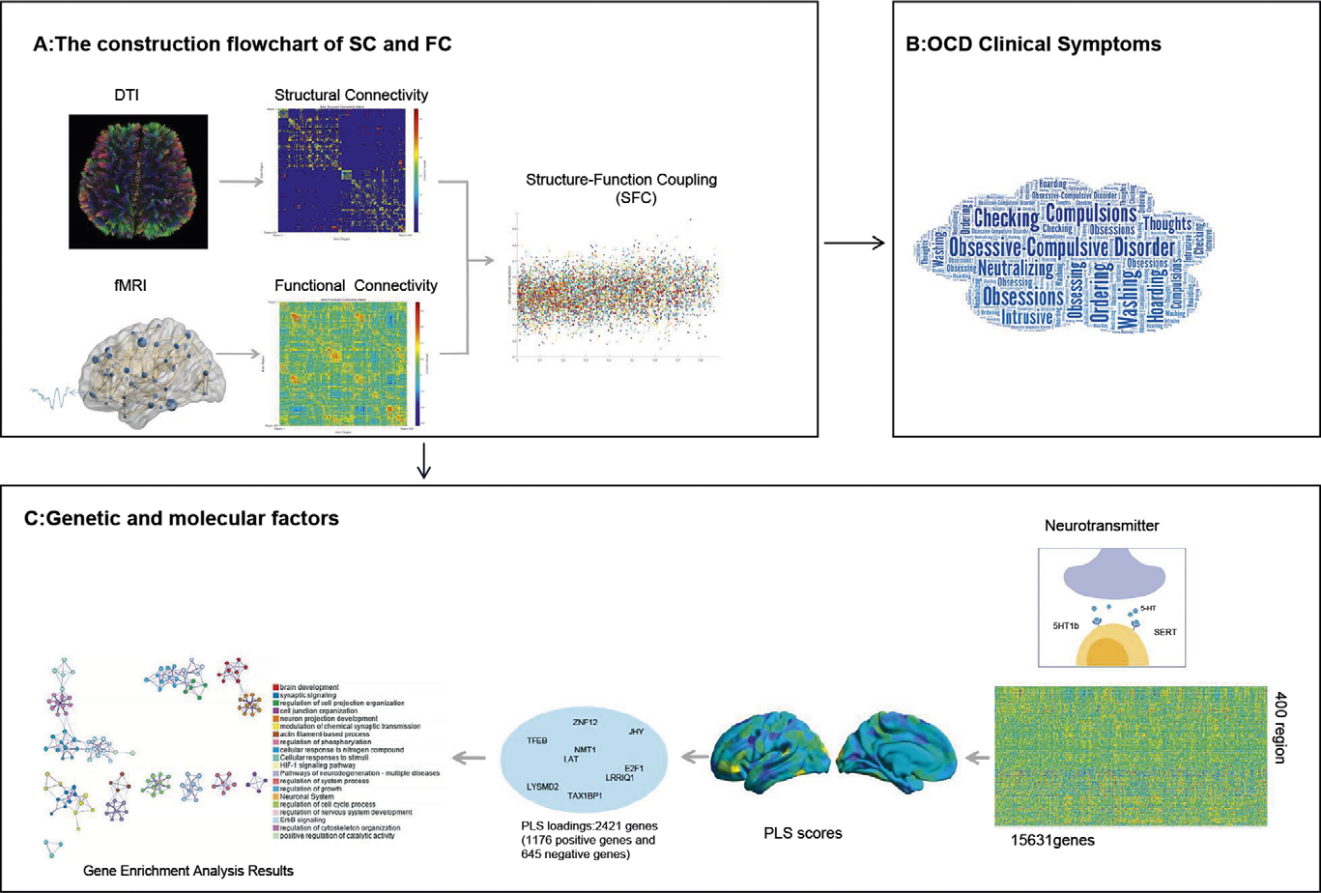
Schematic representation of the multiscale analysis summarizing potential pathways from genes to clinical symptoms. (a) Construction of structural and functional connectivity. Diagram illustrating the computation of structural connectivity (SC) and functional connectivity (FC), along with the quantification of structure–function coupling (SFC). (b) OCD clinical symptoms. Regional SFC alterations (notably in rTPJ) were associated with the severity of compulsive behaviors. (c) Genetic and molecular factors. *Based on the Allen Human Brain Atlas, we identified 2,421 genes associated with SFC abnormalities, which were enriched in brain development, synaptic signaling, and neuronal regulation. Neurotransmitter mapping further suggested a tentative link between SFC alterations and serotonergic receptor distribution. The figure was created using BioGDP.com (Jiang et al., 2025) and WordArt.com, and incorporates elements generated with BrainNet Viewer and DSI Studio.

This study presents a multiscale, exploratory investigation aimed at generating hypotheses about the molecular and neural correlates of abnormal SFC in medication-free OCD. While no significant differences were observed at the whole-brain or network level, exploratory regional analyses revealed alterations in areas, including the rTPJ, which was correlated with the severity of compulsive behaviors (Y-BOCS compulsion subscore) (*β* = 0.218, *p* = 0.033). In particular, the rTPJ simultaneously exhibited alterations in the topological properties of functional networks, highlighting its potential pathological significance. Spatial transcriptomic analysis, leveraging the AHBA, suggested that the abnormal SFC pattern may be associated with genes implicated in neurodevelopmental and synaptic signaling pathways, and revealed tentative spatial correlations with serotonergic signaling. Together, these results provide preliminary, hypothesis-generating evidence for a potential link among transcriptional patterns, SFC abnormalities, and obsessive-compulsive symptoms (Figure 5).

In this study, multiscale network analysis of OCD patients revealed no significant differences at the whole-brain or seven-network level, consistent with Enhancing NeuroImaging Genetics through Meta-Analysis (ENIGMA) findings (Kong et al., 2020). However, we observed reduced SFC in the left temporal lobe, accompanied by a compensatory increase in the upper lateral stripe of the left peripheral visual cortex. These findings align with previous reports of abnormal connectivity in temporal and visual networks in OCD (Ding et al., 2023; Ravindran et al., 2020). Specifically, decreased connectivity in temporal-limbic regions (Reess et al., 2016) and enhanced connectivity involving the visual cortex (Ravindran et al., 2020) have been previously documented. These results collectively support the hypothesis of local network specificity in OCD pathology, whereby neurobiological changes are concentrated in specific subregions rather than following a uniform whole-brain pattern. Notably, this study extends prior work by revealing a visual-temporal compensatory balance and detailed visual subregion abnormalities in medication-free patients, going beyond the coarse-grained seven-network template (Yeo et al., 2011). Future research should validate these findings in larger samples, incorporate dynamic functional connectivity, and explore more detailed network models.

Our study identified that the rTPJ holds critical pathological significance in OCD. This region exhibits both abnormal SFC and alterations in network topological properties (degree centrality and node efficiency), with a significant correlation with the severity of obsessive-compulsive symptoms. This finding is highly consistent with the model of sensory integration-behavioral control circuit dysregulation in OCD. Functionally, the rTPJ is a hub for multisensory integration, coordinating sensory input and behavioral output (Decety & Lamm, 2007), and its dysfunction may contribute to attentional fixation and behavioral rigidity (e.g. repetitive handwashing) in OCD patients. The rTPJ also mediates responses to emotional stimuli (Carter & Huettel, 2013), aligning with the OCD anxiety-obsession cycle mechanism in OCD. Structurally, OCD patients exhibit gray matter thinning (Boedhoe et al., 2018, 2017) and reduced network efficiency (Goodman, Grice, Lapidus, & Coffey, 2014) in the TPJ. Our observed decrease in rTPJ SFC suggests a structure–function mismatch, potentially impairing sensory information integration – consistent with reported visual processing deficits in OCD (Nakao, Okada, & Kanba, 2014). Moreover, the reduced degree centrality and node efficiency indicate a diminished capacity for information integration and communication with other regions, likely exacerbating behavioral inflexibility. In summary, this study highlights the rTPJ as a pivotal node in the sensory-cognitive-behavioral pathway of OCD. Future research should combine transcranial magnetic stimulation (TMS) interventions targeting the rTPJ to verify its regulatory effect on OCD symptoms.

This study further implicates the dysregulation of response inhibition and cognitive control in OCD. As a key component of the PFC, the SMA plays a crucial role in response inhibition and motor planning (Del Casale et al., 2011). Its pathological significance is underscored by its frequent selection as a target in TMS treatment studies for OCD (Rodrigues da Silva et al., 2022; Yu et al., 2022). Consistent with previous studies, our findings indicate that OCD patients exhibit reductions in both gray matter structure and dynamic functional coupling in the right SMA (Ding et al., 2023). This appears to conflict with reports of SMA hyperexcitability as a core deficit in OCD (Koçak, Ceran, Üney, & Hacıyev, 2022). The discrepancy may reflect a vicious cycle of compensatory failure: an initial inhibitory deficit could trigger increased compensatory input to the SMA, but excessive drive may paradoxically lead to over-excitation and weakened functional integration, ultimately exacerbating control failure. Additionally, we found significantly reduced SFC in the right PFC of OCD patients compared with controls. This, together with gray matter atrophy (Reess et al., 2016), white matter microstructural damage, and weakened functional connectivity (Vaghi et al., 2017), forms a multimodal evidence chain, collectively supporting the impairment of structure–function integration in the right PFC. Given the PFC’s central role in cognitive control, such dysfunction may underlie patients’ difficulties in suppressing intrusive thoughts and compulsive behaviors. The critical cross-species role of the PFC in OCD pathology is further highlighted by studies showing that repeated stimulation of the prefrontal–striatal circuit in mice induces OCD-like compulsive behaviors (Ahmari et al., 2013).

Spatial transcriptomic analysis revealed preliminary associations between SFC alterations in the rTPJ and regional gene expression in OCD. Enrichment analysis indicated these genes are primarily involved in neurodevelopment and synaptic signaling – core pathways consistently implicated in the genetics of OCD (Aoki, Hara, & Kunisada, 2017; Haber & Heilbronner, 2013; Slifstein et al., 2015), and resonant with its neurodevelopmental origins in a substantial patient subset (Grassi, Cecchelli, Mazzocato, & Vignozzi, 2021; Nakatani et al., 2011). This molecular profile can be tentatively integrated with established pathophysiological models. Synaptic and neurodevelopmental processes may influence circuit function within the CSTC framework (Haber & Heilbronner, 2013; Song, Wang, Yu, & Lin, 2021), while rTPJ dysfunction could perturb sensory gating into this circuit. Notably, some overlapping genes (e.g. GRID2 and KIT) are also associated with synaptic dysfunction in other neurodevelopmental disorders like autism (Aoki et al., 2017; Yuzaki, 2013), suggesting a potential shared molecular basis for psychiatric comorbidity related to synaptic homeostasis.

This study found that abnormal SFC in the rTPJ showed a preliminary negative association with the regional density of 5-HT₁b receptors and the SERT. This tentatively suggests that increased serotonergic signaling may relate to local structure–function decoupling. This finding can find indirect support from multiple levels. Genetic associations (Taylor, 2013) and evidence that decreased SERT function (linked to polymorphisms like 5-HTTLPR) may lead to synaptic 5-HT accumulation and excessive 5-HT₁b receptor activation, potentially disrupting local circuit balance (Gomes et al., 2018; Kim et al., 2002; Qin et al., 2016). It is critical to view this finding within a broader neurochemical context. Recent resting-state fMRI studies have shown that spontaneous activity in the right superior temporal lobe is associated with multiple receptor systems (e.g. 5-HT₁ₐR, D₂R, mGluR, etc.) (Y. Liu et al., 2023; Murphy et al., 2008), and structural changes – abnormal gray matter morphology in OCD patients – are also linked to the dopamine system. These seemingly inconsistent results collectively indicate that the neurochemical basis of OCD likely involves an imbalance across multiple neurotransmitter systems (Grünblatt, Hauser, & Walitza, 2014). Therefore, the observed spatial association with the 5-HT system in this study should be regarded as a preliminary component of this complex picture, not an exclusive conclusion. This study has several key limitations that must be emphasized, in line with its exploratory nature. Most critically, our gene-level findings are constrained by the AHBA, which has a very small sample size (*n* = 6), includes only the left hemisphere, and exhibits donor heterogeneity. These limitations mean that all transcriptional associations are preliminary and require validation using large-scale, whole-brain transcriptomic data. Additionally, the OCD patients included had anxiety and depression, which may have influenced the results, and the spatial resolution of neurotransmitter atlases is limited. Furthermore, the cross-sectional design of this study precludes any causal inference, so the observed associations cannot establish directionality. Therefore, this work should be considered hypothesis-generating. Future studies with larger, independent samples and multimodal data are needed to verify the proposed pathway from gene expression to neurochemistry, brain coupling and symptoms.

## Conclusion

In summary, this study delves into the neurobiological mechanisms of OCD through a multimodal approach. Neuroimaging reveals functional dysregulation in the brain networks of OCD patients, while transcriptomics uncovers the molecular mechanisms that may drive these network changes. The integration of neurotransmitter mapping into the research allows us to further understand these abnormalities from a neurochemical perspective, thereby forming a cross-level biological framework. Through such data integration, we not only reveal the core pathology of OCD but also provide potential biomarkers for future personalized treatments. We identified significant abnormalities in SFC between HCs and medication-free OCD patients. These differences correspond to overlaps in specific gene expressions, suggesting that the pathological mechanisms of OCD may have an imaging-genetic basis. The combination of these gene expressions with neuroimaging highlights the genetic regulatory mechanisms underlying brain structure–function abnormalities in OCD, offering new imaging-genetic perspectives for future genetic imaging studies and potential interventions.

## Supporting information

10.1017/S0033291726103389.sm001 Zhang et al. supplementary material 1 Zhang et al. supplementary material

10.1017/S0033291726103389.sm002 Zhang et al. supplementary material 2 Zhang et al. supplementary material

## Data Availability

Neuroimaging data preprocessing was completed with freely available software (http://rfmri.org/DPABI). Human gene expression data were sourced from the Allen Brain Atlas (https://human.brain-map.org/static/download). The gene expression data of Allen Brain Atlas was analyzed using abagen (https://github.com/rmarkello/abagen). PLS analysis was carried out using codes available on GitHub (https://github.com/SarahMorgan/Morphometric_Similarity_SZ). JuSpace was employed to perform spatial correlation analyses between magnetic resonance imaging data and neurotransmitter maps derived from nuclear imaging (https://github.com/juryxy/JuSpace). Biological function enrichment analysis was performed via the WebGestalt online tool (https://www.webgestalt.org/).

## Supplementary material

The supplementary material for this article can be found at http://doi.org/10.1017/S0033291726103389.
