# Supplementary material for: Genetic and molecular factors associated with changes in structural-functional coupling in medication-free obsessive-compulsive disorder
Source: Psychol Med. 2026 Feb 20;56:e50. doi: 10.1017/S0033291726103389 (PMC12969207; doi:10.1017/S0033291726103389)
Supplement: Zhang et al. supplementary material 1 — Zhang et al. supplementary material [file S0033291726103389sup001.docx]

1. Supplemental Methods and Materials

1.1. Participants

A total of 100 patients were recruited from the outpatient and inpatient departments of the obsessive-compulsive disorder clinic at the Affiliated Brain Hospital of Nanjing Medical University. The diagnoses of OCD were ascertained by experienced psychiatrists based on the DSM-V using unstructured clinical interviews. For patients, the inclusion criteria were: (1) primary diagnosis of OCD by an experienced psychiatrist according to the Diagnostic and Statistical Manual of Mental Disorders, fifth edition (DSM-V), (2) Have not undergone systematic pharmacotherapy for OCD either in the previous medical history or within the most recent period of 8 weeks or longer,(3) Have not received psychotherapy targeting OCD within the past month. (The cases with only one or two sessions of psychological counseling are not included.The exclusion criteria were: (1) current comorbid schizophrenia and bipolar disorder, (2) neurological disorders and severe somatic disorders, (3) pregnancy and/or breastfeeding, (4) severe suicidal self-injurious behavior or at risk of suicide attempts. Additionally, 90 healthy controls (HCs) matched in terms of sex, and age were recruited via convenience sampling from work, university settings, and the community at large.

1.2. Clinical assessment

1. Yale-Brown Obsessive Compulsive Scale (Y-BOCS): The severity of OCD symptoms was measured using the semi-structured clinician-administered Yale-Brown Obsessive Compulsive Scale, which consists of 10 items。
2. Mini-International Neuropsychiatric Interview (MINI): The MINI is a structured diagnostic interview tool designed for the rapid assessment of common psychiatric disorders according to DSM and ICD criteria. Developed by Sheehan et al., it provides a brief yet reliable diagnostic method for clinical research and epidemiological surveys..
3. Obsessive-Compulsive Inventory-Revised (OCI-R): This is a brief 18-item self-report questionnaire measuring symptoms across six subscales: Washing, Checking, Neutralizing, Obsessing, Ordering, and Hoarding. Each item is rated on a scale from 0 ("Not at all") to 4 ("Extremely"). Higher total scores indicate greater severity of obsessive-compulsive symptoms
4. Beck Anxiety Inventory (BAI) and Beck Depression Inventory-II (BDI-II): Anxiety and depressive symptoms were assessed using the Beck Anxiety Inventory (BAI) and the Beck Depression Inventory-Second Edition (BDI-II) [26], respectively. The BAI includes 21 anxiety symptoms rated on a 0-3 scale, with a total score ranging from 0 to 63. The BDI-II is a 21-item self-report questionnaire using a 4-point scale to reflect symptoms experienced over the past two weeks, with total scores ranging from 0 to 63. For both instruments, higher scores indicate more severe symptoms.
5. Sheehan Disability Scale (SDS): The SDS is a self-report questionnaire designed to assess the impact of mental health problems on an individual's daily functioning. Each of its three domains is typically rated on a 0 to 10 scale, where 0 represents no impairment and 10 represents extreme, incapacitating impairment. A higher total score indicates greater functional disability .

1.3. fMRI Data Acquisition and Preprocessing

Resting-state MR images of the entire brain were acquired on a Siemens 3.0 T scanner at the Department of Radiology, the Affiliated Brain Hospital of Nanjing Medical University. All HCs and OCD patients underwent MRI scans. The rs-fMRI data were acquired using an echo-planar imaging (EPI) sequence with the following parameters: repetition time (TR) = 2000 ms, echo time (TE) = 40 ms, field of view (FOV) = 240 × 240 mm, matrix = 64 × 64, flip angle (FA) = 90°, 36 slices, slice thickness = 4 mm, spacing between slices = 4 mm, and 240 volumes.T1-weighted anatomical images were obtained via an echo-planar imaging sequence (parameters: TR = 1900 ms, TE = 2.48 ms, FOV = 240 × 240 mm, matrix = 256 × 256, FA = 9°, 36 slices, slice thickness = 1 mm, spacing between slices = 0.5 mm, and 240 volumes). DTI scanning: Diffusion imaging with a b-value of 1000 s/mm² and in 30 directions. (parameters: TR = 6600 ms, TE = 93 ms, FOV = 240 × 240 mm, matrix = 128 × 128, 45 slices, slice thickness = 3 mm, spacing between slices = 0 mm). Preprocessing involves several key processing steps using the DPARSF V3.0 toolbox within the SPM8 toolkit. The main steps are as follows:1. The first 10 time points of the images were removed to avoid unstable signals caused by the magnetic field not reaching equilibrium at the beginning of the scan. 2. Since the scanner performed layer-by-layer scanning, there was a time interval for each scan, and the scanning order varied by machine. Therefore, the remaining time layers were corrected with reference to the middle layer to adjust for time differences between layers.3. The remaining images were registered to the first image to correct for head movement.4. Scalp structures were removed to reduce the influence of irrelevant structures during registration, thereby improving registration accuracy.5. The T1-weighted images were aligned to the functional images.6. Linear drift was removed.7. Head movement parameters were adjusted using the Friston 24-parameter model.8. Noise signals from white matter and cerebrospinal fluid were removed: the WM/CSF signals were averaged separately to obtain two average time series as covariates for removal.9. Standardization was performed: since individual brains have differences, it was necessary to standardize them to the same space for comparison, using [3 3 3], which resampled the images to 3 cubic millimeters to match the ROI template.10. To reduce inconvenience in comparative experiments due to structural differences in subjects' brains, each subject's images were registered to the MNI standard space through affine transformation using SPM's standard EPI template.11. A time bandpass filter (0.01-0.1 Hz) was applied to reduce the effects of low-frequency drift and high-frequency noise.12. The images were smoothed to reduce registration inaccuracies and improve the signal-to-noise ratio, with smoothing parameters FWHM set to [3 3 3].

Constructing structural brain networks involves using DSI Studio, a software tool for white matter fiber tractography that can map brain connectivity. It implements several diffusion MRI methods, including DTI, generalized q-sampling imaging (GQI), q-space diffeomorphic reconstruction (QSDR), diffusion MRI connectometry, and generalized deterministic fiber tracking. The main processing steps include correcting for head motion, eddy currents, and gradient directions.After setting the image threshold to 84, a brain mask was obtained, and head motion correction was performed. Fiber tracking was stopped when the threshold was set to FA < 0.2, resulting in an FA map for each subject. Whole-brain fiber tracking was then conducted, and the results were matched to the Schaefer400 atlas.

1.4.Construction of structural-functional coupling

The whole-brain time series was extracted from the preprocessed fMRI data, and the correlation coefficient was calculated using Pearson. The correlation z value was converted to the Fisher coefficient, and the functional network connection matrix was constructed using the Schaefer400 atlas. The structural imaging data was matched to the Schaefer400 atlas through whole-brain fiber tracking, and the number of fibers was calculated to obtain the fiber number matrix. Before constructing the structural-functional connection coupling, the network was sparsed to screen more important connections. The retained connections were recorded as the original weights, and the unretained connections were recorded as 0. For each subject, the connections retained in the structural and functional brain networks after sparseness were spatially one-to-one, that is, the binary network matrix of the structural brain network was equal to the binary network matrix of the functional brain network. Secondly, the structural connections retained in the sparse structural brain network were extracted and converted into vectors, and the functional connections retained in the sparse functional brain network were extracted and converted into another vector. And perform Fisher Z transformation. Finally, calculate the Pearson correlation of the above two vectors for each subject, define the correlation value as the structural-functional connection coupling at the connection level, and calculate a structural-functional connection coupling value at the connection level for each subject.The Schaefer400 atlas is divided into 7 functional subnetworks. These subnetworks are called visual network, somatomotor network, dorsal attention network, salience network, ventral attention network, limbic network, frontoparietal control network, and default mode network.

1.5.Spatial correlation between abnormal structure-function coupling and neurotransmitter systems

These neurotransmitter maps were obtained from PET/SPECT studies and included 5-HT1a (5-hydroxytryptamine receptor 1a subtype), 5-HT1b (5-HT 1b subtype), 5-HT2a (5-HT 2a subtype), 5HT4 (5-HT 4 subtype), D1 (dopamine D1), D2 (dopamine D2), DAT (dopamine transporter), F-DOPA (dopamine synthesis capacity), GABAa (gamma-aminobutyric acid), NAT (norepinephrine transporter), SERT (serotonin transporter), CB1 (cannabinoid receptor 1), CBF_ASL_MRI (measurement of cerebral blood flow), and MRI-induced cerebral hypertension. MRI technology), KappaOp (κ opioid receptor), MU (μ opioid receptor), NMDA (N-methyl-D-aspartate receptor), VAChT (acetylcholine transporter), mGluR5 (metabolic glutamate receptor 5).

1. Supplemental Results
   1. Demographic Characteristics of Participant

All participants self-reported their ethnicity. The sample was predominantly Han Chinese (96.84%), with a small proportion of participants from other ethnic minorities (3.16%). Statistical analysis indicated no significant differences in ethnic composition between the patient and healthy control groups (p > 0.05).Among the 190 participants who completed fMRI scanning, 187 were right-handed (98.42%), and 3 were left-handed or mixed-handed (1.58%).After excluding 10 participants due to excessive head motion (>3 mm), the remaining 180 participants included 180 right-handed and 0 left- or mixed-handed individuals.

- 1. Tables

Table S1. Demographics, clinical measures, and head rotation parameters of HC and OCD subjects：data are presented as the mean±standard deviation(SD). The clinical characteristics between HC and OCD groups were compared by using two independent samples t-test. Gender and Ethnicity is analyzed using the χ2 test.*p<0.05.OCD, obsessive-compulsive disorder ;HC, healthy controls. YBOCS, Yale–Brown Obsessive Compulsive Scale. OCI-R, the Obsessive-Compulsive Inventory-Revised. BAI, the Beck Anxiety Inventory. BDI-II, the Beck Depression Inventory-Second Edition. SDS, the Sheehan Disability Scale.

| Items | HC  (n=90) | OCD  (n=100) | T values |
| --- | --- | --- | --- |
| gender(male/female) | 39/51 | 57/43 | 3.539 |
| Age (years) | 26.50±6.13 | 26.18±7.49 | -1.554 |
| Education level(years) | 16.70±2.36 | 14.57±3.42 | 20.097* |
| YBOCS | 0 | 23.21±6.26 | 130.869* |
| Obsession | 0 | 11.79±3.36 | 180.938* |
| Compulsion | 0 | 11.42±3.67 | 127.423* |
| OCIR | 0 | 25.7±11.63 | 156.477* |
| BDI | 0.29±2.74 | 16.59±9.87 | 115.100* |
| BAI | 0.14±1.37 | 15.9±10.29 | 174.083* |
| SDS | 0 | 8.83±7.33 | 205.024* |
| Ethnicity(Han/others) | 87/3 | 97/3 | 0.017 |

Table S2.Substance Use of Participants: Zero potential participants were excluded due to a current substance use disorder (SUD) as assessed by the MINI.

| Substance Type | Current User | Past Users |
| --- | --- | --- |
| Alcoho | 0 | 0 |
| Cannabis | 0 | 0 |
| Tobacco | 0 | 0 |
| Other Drugs | 0 | 0 |

Table S3. Between-Group Effects on Clinical Measures

| Source | Dependent Variable | F | p |
| --- | --- | --- | --- |
| Group | YBOCS | 1173.796 | <.001 |
|  | Obsessive Thoughts | 1050.095 | <.001 |
|  | Compulsive Behaviors | 922.444 | <.001 |
|  | OCI-R | 332.043 | <.001 |
|  | Washing | 115.963 | <.001 |
|  | Obsessing | 301.21 | <.001 |
|  | Hoarding | 85.963 | <.001 |
|  | Ordering | 103.187 | <.001 |
|  | Checking | 193.911 | <.001 |
|  | Mental Neutralizing | 87.601 | <.001 |
|  | BDI | 190.426 | <.001 |
|  | BAI | 168.407 | <.001 |
|  | SDS | 307.264 | <.001 |

Table S4.Results of Whole-Brain, Within-Network, and Between-Network Structural-Functional Coupling

|  | | **t** | ***P*** |
| --- | --- | --- | --- |
| **The whole brain** | | -1.439 | 0.152 |
| **Within modular interaction** | | |  |
| VIS |  | -0.240 | 1.000 |
| SMN |  | 0.359 | 1.000 |
| DAN |  | -1.499 | 1.000 |
| VAN |  | -1.827 | 1.000 |
| LIM |  | -0.175 | 1.000 |
| FPN |  | -1.921 | 1.000 |
| DMN |  | -1.004 | 1.000 |
| Between modular interaction | | |  |
| Modular 1 | Modular 2 |  |  |
| VIS | SMN | -2.310 | 0.617 |
|  | DAN | -0.470 | 1.000 |
|  | VAN | -0.836 | 1.000 |
|  | LIM | -0.151 | 1.000 |
|  | FPN | -0.011 | 1.000 |
|  | DMN | -0.301 | 1.000 |
| SMN | DAN | -1.833 | 1.000 |
|  | VAN | -0.989 | 1.000 |
|  | LIM | -0.728 | 1.000 |
|  | FPN | -0.689 | 1.000 |
|  | DMN | -0.910 | 1.000 |
| DAN | VAN | -0.099 | 1.000 |
|  | LIM | 1.459 | 1.000 |
|  | FPN | 0.460 | 1.000 |
|  | DMN | 1.888 | 1.000 |
| VAN | LIM | -0.465 | 1.000 |
|  | FPN | -1.154 | 1.000 |
|  | DMN | -0.809 | 1.000 |
| LIM | FPN | -0.623 | 1.000 |
|  | DMN | -1.367 | 1.000 |
| FPN | DMN | -0.595 | 1.000 |

* p<0.05

Table S5.Results of Structural-Functional Coupling in 400 Brain Regions

|  | **Couping** |  |
| --- | --- | --- |
|  | **T_Statistic** | **P_Value** |
| Left_VisCent_ExStr_1 | 2.4013 | 1.0000 |
| Left_VisCent_ExStr_2 | 0.6618 | 1.0000 |
| Left_VisCent_ExStr_3 | 2.0998 | 1.0000 |
| Left_VisCent_ExStr_4 | -0.1380 | 1.0000 |
| Left_VisCent_ExStr_5 | 0.2394 | 1.0000 |
| Left_VisCent_ExStr_6 | -0.3117 | 1.0000 |
| Left_VisCent_ExStr_7 | 0.0363 | 1.0000 |
| Left_VisCent_ExStr_8 | -0.0408 | 1.0000 |
| Left_VisCent_ExStr_9 | -0.9966 | 1.0000 |
| Left_VisCent_ExStr_10 | -1.5988 | 1.0000 |
| Left_VisCent_ExStr_11 | 0.1525 | 1.0000 |
| Left_VisCent_ExStr_12 | -0.2514 | 1.0000 |
| Left_VisPeri_ExStrSup_1 | -1.5480 | 1.0000 |
| Left_VisPeri_ExStrSup_2 | 0.7122 | 1.0000 |
| Left_VisPeri_ExStrSup_3 | 1.1009 | 1.0000 |
| Left_VisPeri_ExStrSup_4 | -0.3338 | 1.0000 |
| Left_VisPeri_ExStrSup_5 | 1.0436 | 1.0000 |
| Left_VisPeri_ExStrSup_6 | 0.3966 | 1.0000 |
| Left_VisPeri_ExStrSup_7 | -0.7918 | 1.0000 |
| Left_VisPeri_ExStrSup_8 | -0.6993 | 1.0000 |
| Left_VisPeri_ExStrSup_9 | -2.3182 | 1.0000 |
| Left_VisPeri_ExStrSup_10 | -0.4260 | 1.0000 |
| Left_VisPeri_ExStrSup_11 | 0.5788 | 1.0000 |
| Left_VisPeri_ExStrSup_12 | 5.0692 | 0.0004 |
| Left_SomMotA_1 | 0.5411 | 1.0000 |
| Left_SomMotA_2 | -1.5002 | 1.0000 |
| Left_SomMotA_3 | 1.6534 | 1.0000 |
| Left_SomMotA_4 | 2.0814 | 1.0000 |
| Left_SomMotA_5 | 2.1937 | 1.0000 |
| Left_SomMotA_6 | -0.9056 | 1.0000 |
| Left_SomMotA_7 | 0.9728 | 1.0000 |
| Left_SomMotA_8 | 0.6521 | 1.0000 |
| Left_SomMotA_9 | -0.9693 | 1.0000 |
| Left_SomMotA_10 | -1.5405 | 1.0000 |
| Left_SomMotA_11 | 1.6773 | 1.0000 |
| Left_SomMotA_12 | -0.1095 | 1.0000 |
| Left_SomMotA_13 | 5.1446 | 0.0003 |
| Left_SomMotA_14 | 0.4205 | 1.0000 |
| Left_SomMotA_15 | 0.4217 | 1.0000 |
| Left_SomMotA_16 | -0.2780 | 1.0000 |
| Left_SomMotA_17 | -0.4571 | 1.0000 |
| Left_SomMotA_18 | 0.5561 | 1.0000 |
| Left_SomMotA_19 | 1.7400 | 1.0000 |
| Left_SomMotB_Aud_1 | -0.5302 | 1.0000 |
| Left_SomMotB_Aud_2 | -1.7252 | 1.0000 |
| Left_SomMotB_Aud_3 | -2.4703 | 1.0000 |
| Left_SomMotB_Aud_4 | -1.1395 | 1.0000 |
| Left_SomMotB_Aud_5 | -0.3716 | 1.0000 |
| Left_SomMotB_Aud_6 | 0.8944 | 1.0000 |
| Left_SomMotB_Aud_7 | 1.4226 | 1.0000 |
| Left_SomMotB_Aud_8 | 1.5642 | 1.0000 |
| Left_SomMotB_Aud_9 | 0.9708 | 1.0000 |
| Left_SomMotB_Aud_10 | -1.4899 | 1.0000 |
| Left_SomMotB_Aud_11 | 0.3219 | 1.0000 |
| Left_SomMotB_Aud_12 | 0.1417 | 1.0000 |
| Left_SomMotB_Aud_13 | -1.3177 | 1.0000 |
| Left_SomMotB_Aud_14 | -0.2942 | 1.0000 |
| Left_SomMotB_Aud_15 | -1.4516 | 1.0000 |
| Left_SomMotB_Aud_16 | 0.3607 | 1.0000 |
| Left_DorsAttnA_TempOcc_1 | 1.6672 | 1.0000 |
| Left_DorsAttnA_TempOcc_2 | -0.9991 | 1.0000 |
| Left_DorsAttnA_TempOcc_3 | 0.9319 | 1.0000 |
| Left_DorsAttnA_TempOcc_4 | 2.0376 | 1.0000 |
| Left_DorsAttnA_ParOcc_1 | -1.1603 | 1.0000 |
| Left_DorsAttnA_SPL_1 | -1.7781 | 1.0000 |
| Left_DorsAttnA_SPL_2 | 3.0342 | 1.0000 |
| Left_DorsAttnA_SPL_3 | 0.1844 | 1.0000 |
| Left_DorsAttnA_SPL_4 | -0.3447 | 1.0000 |
| Left_DorsAttnA_SPL_5 | 2.7199 | 1.0000 |
| Left_DorsAttnA_SPL_6 | 5.9568 | 0.0000 |
| Left_DorsAttnA_SPL_7 | -0.3501 | 1.0000 |
| Left_DorsAttnA_SPL_8 | 0.1243 | 1.0000 |
| Left_DorsAttnB_PostC_1 | 0.1333 | 1.0000 |
| Left_DorsAttnB_PostC_2 | -0.4055 | 1.0000 |
| Left_DorsAttnB_PostC_3 | -0.1672 | 1.0000 |
| Left_DorsAttnB_PostC_4 | -3.2949 | 0.4767 |
| Left_DorsAttnB_PostC_5 | 0.4221 | 1.0000 |
| Left_DorsAttnB_PostC_6 | -1.6030 | 1.0000 |
| Left_DorsAttnB_PostC_7 | -1.3178 | 1.0000 |
| Left_DorsAttnB_PostC_8 | -1.1345 | 1.0000 |
| Left_DorsAttnB_PostC_9 | -0.9091 | 1.0000 |
| Left_DorsAttnB_FEF_1 | -0.2849 | 1.0000 |
| Left_DorsAttnB_FEF_2 | 0.0834 | 1.0000 |
| Left_DorsAttnB_FEF_3 | -1.4718 | 1.0000 |
| Left_DorsAttnB_PrCv_1 | 0.2088 | 1.0000 |
| Left_SalVentAttnA_ParOper_1 | 0.3279 | 1.0000 |
| Left_SalVentAttnA_ParOper_2 | 0.0695 | 1.0000 |
| Left_SalVentAttnA_ParOper_3 | 1.9407 | 1.0000 |
| Left_SalVentAttnA_Ins_1 | 0.8470 | 1.0000 |
| Left_SalVentAttnA_Ins_2 | -1.8725 | 1.0000 |
| Left_SalVentAttnA_Ins_3 | -0.6883 | 1.0000 |
| Left_SalVentAttnA_Ins_4 | -0.5082 | 1.0000 |
| Left_SalVentAttnA_Ins_5 | -1.1013 | 1.0000 |
| Left_SalVentAttnA_Ins_6 | 0.0409 | 1.0000 |
| Left_SalVentAttnA_ParMed_1 | -0.4832 | 1.0000 |
| Left_SalVentAttnA_ParMed_2 | 1.1493 | 1.0000 |
| Left_SalVentAttnA_ParMed_3 | 1.5531 | 1.0000 |
| Left_SalVentAttnA_FrMed_1 | -1.6109 | 1.0000 |
| Left_SalVentAttnA_FrMed_2 | 1.6715 | 1.0000 |
| Left_SalVentAttnA_FrMed_3 | 1.1606 | 1.0000 |
| Left_SalVentAttnB_PFCl_1 | 0.3238 | 1.0000 |
| Left_SalVentAttnB_PFCl_2 | 0.3684 | 1.0000 |
| Left_SalVentAttnB_PFCl_3 | 1.3264 | 1.0000 |
| Left_SalVentAttnB_PFCv_1 | -0.4115 | 1.0000 |
| Left_SalVentAttnB_PFCv_2 | 0.6639 | 1.0000 |
| Left_SalVentAttnB_PFCv_3 | 2.1639 | 1.0000 |
| Left_SalVentAttnB_OFC_1 | -1.8516 | 1.0000 |
| Left_SalVentAttnB_PFCmp_1 | -0.2679 | 1.0000 |
| Left_Limbic_OFC_1 | 0.1943 | 1.0000 |
| Left_Limbic_OFC_2 | -0.3796 | 1.0000 |
| Left_Limbic_OFC_3 | 2.3196 | 1.0000 |
| Left_Limbic_OFC_4 | 1.2055 | 1.0000 |
| Left_Limbic_OFC_5 | -2.2502 | 1.0000 |
| Left_Limbic_TempPole_1 | 1.4012 | 1.0000 |
| Left_Limbic_TempPole_2 | 0.1889 | 1.0000 |
| Left_Limbic_TempPole_3 | -0.0878 | 1.0000 |
| Left_Limbic_TempPole_4 | 1.5713 | 1.0000 |
| Left_Limbic_TempPole_5 | -1.6090 | 1.0000 |
| Left_Limbic_TempPole_6 | -1.1698 | 1.0000 |
| Left_Limbic_TempPole_7 | -1.1034 | 1.0000 |
| Left_ContA_Temp_1 | -0.9298 | 1.0000 |
| Left_ContA_IPS_1 | 1.3402 | 1.0000 |
| Left_ContA_IPS_2 | -0.1487 | 1.0000 |
| Left_ContA_IPS_3 | 0.4977 | 1.0000 |
| Left_ContA_IPS_4 | 0.0577 | 1.0000 |
| Left_ContA_IPS_5 | -0.4285 | 1.0000 |
| Left_ContA_PFCd_1 | -0.6411 | 1.0000 |
| Left_ContA_PFCl_1 | 1.1857 | 1.0000 |
| Left_ContA_PFCl_2 | 1.2933 | 1.0000 |
| Left_ContA_PFCl_3 | 1.1319 | 1.0000 |
| Left_ContA_PFCl_4 | 0.5578 | 1.0000 |
| Left_ContA_PFCl_5 | -1.4267 | 1.0000 |
| Left_ContA_Cinga_1 | 1.0240 | 1.0000 |
| Left_ContB_Temp_1 | 0.7806 | 1.0000 |
| Left_ContB_Temp_2 | 2.4549 | 1.0000 |
| Left_ContB_IPL_1 | 0.4664 | 1.0000 |
| Left_ContB_IPL_2 | 0.4442 | 1.0000 |
| Left_ContB_IPL_3 | 1.0367 | 1.0000 |
| Left_ContB_PFCd_1 | -0.5352 | 1.0000 |
| Left_ContB_PFClv_1 | -0.8916 | 1.0000 |
| Left_ContB_PFClv_2 | -3.9228 | 0.0502 |
| Left_ContB_PFClv_3 | -0.6162 | 1.0000 |
| Left_ContB_PFCmp_1 | -1.3523 | 1.0000 |
| Left_ContC_pCun_1 | -0.3744 | 1.0000 |
| Left_ContC_pCun_2 | 0.2283 | 1.0000 |
| Left_ContC_pCun_3 | -0.1186 | 1.0000 |
| Left_ContC_Cingp_1 | 0.5628 | 1.0000 |
| Left_ContC_Cingp_2 | 0.4539 | 1.0000 |
| Left_DefaultA_IPL_1 | -0.0978 | 1.0000 |
| Left_DefaultA_IPL_2 | -1.3912 | 1.0000 |
| Left_DefaultA_PFCd_1 | 0.3354 | 1.0000 |
| Left_DefaultA_PFCd_2 | -2.1263 | 1.0000 |
| Left_DefaultA_PFCd_3 | 1.4480 | 1.0000 |
| Left_DefaultA_PCC_1 | -0.6891 | 1.0000 |
| Left_DefaultA_PCC_2 | 0.0038 | 1.0000 |
| Left_DefaultA_PCC_3 | -1.6727 | 1.0000 |
| Left_DefaultA_PCC_4 | -0.2825 | 1.0000 |
| Left_DefaultA_PCC_5 | 1.3668 | 1.0000 |
| Left_DefaultA_PCC_6 | 0.0512 | 1.0000 |
| Left_DefaultA_PCC_7 | -0.2631 | 1.0000 |
| Left_DefaultA_PFCm_1 | 1.8700 | 1.0000 |
| Left_DefaultA_PFCm_2 | 1.4315 | 1.0000 |
| Left_DefaultA_PFCm_3 | 0.9001 | 1.0000 |
| Left_DefaultA_PFCm_4 | -0.9617 | 1.0000 |
| Left_DefaultA_PFCm_5 | 2.2408 | 1.0000 |
| Left_DefaultA_PFCm_6 | -0.2664 | 1.0000 |
| Left_DefaultB_Temp_1 | 3.9103 | 0.0527 |
| Left_DefaultB_Temp_2 | -0.5519 | 1.0000 |
| Left_DefaultB_Temp_3 | 0.3123 | 1.0000 |
| Left_DefaultB_Temp_4 | 5.7402 | 0.0000 |
| Left_DefaultB_Temp_5 | 0.6989 | 1.0000 |
| Left_DefaultB_Temp_6 | 2.1302 | 1.0000 |
| Left_DefaultB_IPL_1 | 1.0297 | 1.0000 |
| Left_DefaultB_IPL_2 | 0.4801 | 1.0000 |
| Left_DefaultB_PFCd_1 | 2.4175 | 1.0000 |
| Left_DefaultB_PFCd_2 | 0.1166 | 1.0000 |
| Left_DefaultB_PFCd_3 | 0.6467 | 1.0000 |
| Left_DefaultB_PFCd_4 | -0.2687 | 1.0000 |
| Left_DefaultB_PFCd_5 | -0.3233 | 1.0000 |
| Left_DefaultB_PFCd_6 | 0.9397 | 1.0000 |
| Left_DefaultB_PFCl_1 | -0.9545 | 1.0000 |
| Left_DefaultB_PFCl_2 | 2.0063 | 1.0000 |
| Left_DefaultB_PFCv_1 | 0.1630 | 1.0000 |
| Left_DefaultB_PFCv_2 | -0.1321 | 1.0000 |
| Left_DefaultB_PFCv_3 | 0.4082 | 1.0000 |
| Left_DefaultB_PFCv_4 | -1.5397 | 1.0000 |
| Left_DefaultB_PFCv_5 | 0.6729 | 1.0000 |
| Left_DefaultC_IPL_1 | -3.0540 | 1.0000 |
| Left_DefaultC_Rsp_1 | 1.6706 | 1.0000 |
| Left_DefaultC_Rsp_2 | -1.7472 | 1.0000 |
| Left_DefaultC_Rsp_3 | -0.5256 | 1.0000 |
| Left_DefaultC_PHC_1 | 0.5758 | 1.0000 |
| Left_DefaultC_PHC_2 | -0.5007 | 1.0000 |
| Left_DefaultC_PHC_3 | 0.4660 | 1.0000 |
| Left_TempPar_1 | 0.2944 | 1.0000 |
| Left_TempPar_2 | -0.0754 | 1.0000 |
| Left_TempPar_3 | 1.4396 | 1.0000 |
| Left_TempPar_4 | -0.8958 | 1.0000 |
| Left_TempPar_5 | -0.1889 | 1.0000 |
| Left_TempPar_6 | -1.6006 | 1.0000 |
| Right_VisCent_ExStr_1 | 2.4266 | 1.0000 |
| Right_VisCent_ExStr_2 | 0.6554 | 1.0000 |
| Right_VisCent_ExStr_3 | 2.0350 | 1.0000 |
| Right_VisCent_ExStr_4 | 0.6807 | 1.0000 |
| Right_VisCent_ExStr_5 | -0.0581 | 1.0000 |
| Right_VisCent_ExStr_6 | -1.4087 | 1.0000 |
| Right_VisCent_ExStr_7 | -1.4377 | 1.0000 |
| Right_VisCent_ExStr_8 | 2.0053 | 1.0000 |
| Right_VisCent_ExStr_9 | -0.6853 | 1.0000 |
| Right_VisCent_ExStr_10 | -0.0459 | 1.0000 |
| Right_VisCent_ExStr_11 | 0.7797 | 1.0000 |
| Right_VisCent_ExStr_12 | -2.3591 | 1.0000 |
| Right_VisPeri_ExStrSup_1 | 0.6441 | 1.0000 |
| Right_VisPeri_ExStrSup_2 | 0.7335 | 1.0000 |
| Right_VisPeri_ExStrSup_3 | 1.0691 | 1.0000 |
| Right_VisPeri_ExStrSup_4 | 1.5545 | 1.0000 |
| Right_VisPeri_ExStrSup_5 | 0.1923 | 1.0000 |
| Right_VisPeri_ExStrSup_6 | 0.6945 | 1.0000 |
| Right_VisPeri_ExStrSup_7 | -0.7448 | 1.0000 |
| Right_VisPeri_ExStrSup_8 | -2.1785 | 1.0000 |
| Right_VisPeri_ExStrSup_9 | 2.2143 | 1.0000 |
| Right_VisPeri_ExStrSup_10 | 1.2053 | 1.0000 |
| Right_VisPeri_ExStrSup_11 | 0.8299 | 1.0000 |
| Right_SomMotA_1 | -0.8946 | 1.0000 |
| Right_SomMotA_2 | 0.2325 | 1.0000 |
| Right_SomMotA_3 | 0.8635 | 1.0000 |
| Right_SomMotA_4 | 0.6726 | 1.0000 |
| Right_SomMotA_5 | -1.1930 | 1.0000 |
| Right_SomMotA_6 | 1.2542 | 1.0000 |
| Right_SomMotA_7 | 0.6907 | 1.0000 |
| Right_SomMotA_8 | -0.8044 | 1.0000 |
| Right_SomMotA_9 | -0.7375 | 1.0000 |
| Right_SomMotA_10 | -3.9309 | 0.0487 |
| Right_SomMotA_11 | 0.0978 | 1.0000 |
| Right_SomMotA_12 | 1.4330 | 1.0000 |
| Right_SomMotA_13 | 0.8033 | 1.0000 |
| Right_SomMotA_14 | -1.8029 | 1.0000 |
| Right_SomMotA_15 | -0.5105 | 1.0000 |
| Right_SomMotA_16 | 0.6164 | 1.0000 |
| Right_SomMotA_17 | -0.1234 | 1.0000 |
| Right_SomMotA_18 | 0.1318 | 1.0000 |
| Right_SomMotA_19 | 0.4756 | 1.0000 |
| Right_SomMotA_20 | -2.7525 | 1.0000 |
| Right_SomMotB_S2_1 | -0.0670 | 1.0000 |
| Right_SomMotB_S2_2 | 1.7537 | 1.0000 |
| Right_SomMotB_S2_3 | 0.0656 | 1.0000 |
| Right_SomMotB_S2_4 | -0.9752 | 1.0000 |
| Right_SomMotB_S2_5 | 1.6852 | 1.0000 |
| Right_SomMotB_S2_6 | -0.3502 | 1.0000 |
| Right_SomMotB_S2_7 | 2.2464 | 1.0000 |
| Right_SomMotB_S2_8 | 0.1300 | 1.0000 |
| Right_SomMotB_S2_9 | -0.7798 | 1.0000 |
| Right_SomMotB_S2_10 | 0.7180 | 1.0000 |
| Right_SomMotB_S2_11 | 1.9794 | 1.0000 |
| Right_SomMotB_S2_12 | -0.1531 | 1.0000 |
| Right_SomMotB_S2_13 | -1.8668 | 1.0000 |
| Right_SomMotB_S2_14 | -1.4822 | 1.0000 |
| Right_SomMotB_S2_15 | -1.3630 | 1.0000 |
| Right_DorsAttnA_TempOcc_1 | -1.7607 | 1.0000 |
| Right_DorsAttnA_TempOcc_2 | 1.2317 | 1.0000 |
| Right_DorsAttnA_TempOcc_3 | -1.2251 | 1.0000 |
| Right_DorsAttnA_TempOcc_4 | -2.0129 | 1.0000 |
| Right_DorsAttnA_TempOcc_5 | 1.9503 | 1.0000 |
| Right_DorsAttnA_SPL_1 | -1.8283 | 1.0000 |
| Right_DorsAttnA_SPL_2 | -0.7890 | 1.0000 |
| Right_DorsAttnA_SPL_3 | -1.4143 | 1.0000 |
| Right_DorsAttnA_SPL_4 | 0.7187 | 1.0000 |
| Right_DorsAttnA_SPL_5 | 1.7141 | 1.0000 |
| Right_DorsAttnA_SPL_6 | -3.2242 | 0.6030 |
| Right_DorsAttnA_SPL_7 | 0.0584 | 1.0000 |
| Right_DorsAttnA_SPL_8 | 2.9677 | 1.0000 |
| Right_DorsAttnA_SPL_9 | 1.6962 | 1.0000 |
| Right_DorsAttnB_TempOcc_1 | 1.2717 | 1.0000 |
| Right_DorsAttnB_PostC_1 | 3.3715 | 0.3680 |
| Right_DorsAttnB_PostC_2 | -0.5331 | 1.0000 |
| Right_DorsAttnB_PostC_3 | -1.4804 | 1.0000 |
| Right_DorsAttnB_PostC_4 | 0.0370 | 1.0000 |
| Right_DorsAttnB_PostC_5 | 0.0261 | 1.0000 |
| Right_DorsAttnB_PostC_6 | -0.7532 | 1.0000 |
| Right_DorsAttnB_PostC_7 | 0.4016 | 1.0000 |
| Right_DorsAttnB_PostC_8 | 0.6788 | 1.0000 |
| Right_DorsAttnB_FEF_1 | 0.3931 | 1.0000 |
| Right_DorsAttnB_FEF_2 | 2.9163 | 1.0000 |
| Right_DorsAttnB_FEF_3 | -0.3380 | 1.0000 |
| Right_SalVentAttnA_ParOper_1 | -1.6818 | 1.0000 |
| Right_SalVentAttnA_ParOper_2 | 1.3056 | 1.0000 |
| Right_SalVentAttnA_ParOper_3 | -0.6837 | 1.0000 |
| Right_SalVentAttnA_PrC_1 | 0.3229 | 1.0000 |
| Right_SalVentAttnA_Ins_1 | -0.7986 | 1.0000 |
| Right_SalVentAttnA_Ins_2 | -0.8283 | 1.0000 |
| Right_SalVentAttnA_Ins_3 | 1.7567 | 1.0000 |
| Right_SalVentAttnA_Ins_4 | -0.3920 | 1.0000 |
| Right_SalVentAttnA_Ins_5 | -2.5328 | 1.0000 |
| Right_SalVentAttnA_Ins_6 | 0.2998 | 1.0000 |
| Right_SalVentAttnA_Ins_7 | -0.0973 | 1.0000 |
| Right_SalVentAttnA_ParMed_1 | -0.3853 | 1.0000 |
| Right_SalVentAttnA_ParMed_2 | -3.3321 | 0.4206 |
| Right_SalVentAttnA_ParMed_3 | 1.0964 | 1.0000 |
| Right_SalVentAttnA_ParMed_4 | -2.9414 | 1.0000 |
| Right_SalVentAttnA_ParMed_5 | -1.1018 | 1.0000 |
| Right_SalVentAttnA_ParMed_6 | -2.0478 | 1.0000 |
| Right_SalVentAttnA_ParMed_7 | -0.1094 | 1.0000 |
| Right_SalVentAttnA_ParMed_8 | -1.1001 | 1.0000 |
| Right_SalVentAttnB_IPL_1 | -0.1437 | 1.0000 |
| Right_SalVentAttnB_PFCl_1 | 0.5972 | 1.0000 |
| Right_SalVentAttnB_PFCl_2 | -0.4057 | 1.0000 |
| Right_SalVentAttnB_PFCl_3 | -1.1705 | 1.0000 |
| Right_SalVentAttnB_PFCl_4 | -1.8304 | 1.0000 |
| Right_SalVentAttnB_PFCv_1 | 1.4804 | 1.0000 |
| Right_SalVentAttnB_PFCv_2 | 0.8254 | 1.0000 |
| Right_SalVentAttnB_PFCmp_1 | -1.6116 | 1.0000 |
| Right_SalVentAttnB_PFCmp_2 | -2.1845 | 1.0000 |
| Right_Limbic_OFC_1 | 1.9959 | 1.0000 |
| Right_Limbic_OFC_2 | 0.5916 | 1.0000 |
| Right_Limbic_OFC_3 | -1.1158 | 1.0000 |
| Right_Limbic_OFC_4 | 0.6050 | 1.0000 |
| Right_Limbic_OFC_5 | 1.6511 | 1.0000 |
| Right_Limbic_OFC_6 | -1.4702 | 1.0000 |
| Right_Limbic_TempPole_1 | -0.3512 | 1.0000 |
| Right_Limbic_TempPole_2 | 0.8876 | 1.0000 |
| Right_Limbic_TempPole_3 | 0.1181 | 1.0000 |
| Right_Limbic_TempPole_4 | 0.2417 | 1.0000 |
| Right_Limbic_TempPole_5 | 0.7892 | 1.0000 |
| Right_Limbic_TempPole_6 | 0.6611 | 1.0000 |
| Right_ContA_IPS_1 | -1.4200 | 1.0000 |
| Right_ContA_IPS_2 | 0.3671 | 1.0000 |
| Right_ContA_IPS_3 | 0.2254 | 1.0000 |
| Right_ContA_IPS_4 | 2.6745 | 1.0000 |
| Right_ContA_PFCd_1 | -1.2097 | 1.0000 |
| Right_ContA_PFCl_1 | 1.9048 | 1.0000 |
| Right_ContA_PFCl_2 | -0.0255 | 1.0000 |
| Right_ContA_PFCl_3 | 1.6168 | 1.0000 |
| Right_ContA_PFCl_4 | -0.5165 | 1.0000 |
| Right_ContA_PFCl_5 | -1.5125 | 1.0000 |
| Right_ContA_Cinga_1 | 0.4530 | 1.0000 |
| Right_ContB_Temp_1 | -2.1509 | 1.0000 |
| Right_ContB_Temp_2 | 0.8785 | 1.0000 |
| Right_ContB_IPL_1 | -0.0434 | 1.0000 |
| Right_ContB_IPL_2 | 0.1663 | 1.0000 |
| Right_ContB_IPL_3 | 0.2740 | 1.0000 |
| Right_ContB_IPL_4 | -0.8588 | 1.0000 |
| Right_ContB_PFCld_1 | -3.9990 | 0.0375 |
| Right_ContB_PFCld_2 | 0.5927 | 1.0000 |
| Right_ContB_PFCld_3 | -2.2323 | 1.0000 |
| Right_ContB_PFCld_4 | -0.8683 | 1.0000 |
| Right_ContB_PFClv_1 | 1.5675 | 1.0000 |
| Right_ContB_PFClv_2 | -1.6042 | 1.0000 |
| Right_ContB_PFClv_3 | 1.3881 | 1.0000 |
| Right_ContB_PFClv_4 | 0.6463 | 1.0000 |
| Right_ContB_PFCmp_1 | 1.0302 | 1.0000 |
| Right_ContC_pCun_1 | 0.2014 | 1.0000 |
| Right_ContC_pCun_2 | -2.1238 | 1.0000 |
| Right_ContC_pCun_3 | -2.0056 | 1.0000 |
| Right_ContC_pCun_4 | -1.2496 | 1.0000 |
| Right_ContC_pCun_5 | -2.1382 | 1.0000 |
| Right_ContC_Cingp_1 | -0.0513 | 1.0000 |
| Right_ContC_Cingp_2 | -0.0918 | 1.0000 |
| Right_DefaultA_Temp_1 | -1.9379 | 1.0000 |
| Right_DefaultA_IPL_1 | -0.5667 | 1.0000 |
| Right_DefaultA_IPL_2 | -2.3138 | 1.0000 |
| Right_DefaultA_PFCd_1 | 1.9169 | 1.0000 |
| Right_DefaultA_PFCd_2 | 0.8524 | 1.0000 |
| Right_DefaultA_PCC_1 | 0.0325 | 1.0000 |
| Right_DefaultA_PCC_2 | -0.1230 | 1.0000 |
| Right_DefaultA_PCC_3 | -1.8845 | 1.0000 |
| Right_DefaultA_PCC_4 | 0.6855 | 1.0000 |
| Right_DefaultA_PCC_5 | -0.5372 | 1.0000 |
| Right_DefaultA_PFCm_1 | -0.5484 | 1.0000 |
| Right_DefaultA_PFCm_2 | -1.5999 | 1.0000 |
| Right_DefaultA_PFCm_3 | 2.2150 | 1.0000 |
| Right_DefaultA_PFCm_4 | 0.9789 | 1.0000 |
| Right_DefaultA_PFCm_5 | -0.8542 | 1.0000 |
| Right_DefaultA_PFCm_6 | 0.4354 | 1.0000 |
| Right_DefaultB_Temp_1 | -1.3438 | 1.0000 |
| Right_DefaultB_Temp_2 | 0.1424 | 1.0000 |
| Right_DefaultB_AntTemp_1 | 0.0150 | 1.0000 |
| Right_DefaultB_PFCd_1 | -0.8081 | 1.0000 |
| Right_DefaultB_PFCd_2 | -0.1238 | 1.0000 |
| Right_DefaultB_PFCd_3 | 1.7477 | 1.0000 |
| Right_DefaultB_PFCd_4 | -0.1854 | 1.0000 |
| Right_DefaultB_PFCd_5 | -0.2199 | 1.0000 |
| Right_DefaultB_PFCv_1 | -0.9880 | 1.0000 |
| Right_DefaultB_PFCv_2 | -0.5215 | 1.0000 |
| Right_DefaultB_PFCv_3 | -0.6879 | 1.0000 |
| Right_DefaultC_IPL_1 | 2.6804 | 1.0000 |
| Right_DefaultC_IPL_2 | -0.1875 | 1.0000 |
| Right_DefaultC_Rsp_1 | -1.9024 | 1.0000 |
| Right_DefaultC_Rsp_2 | 1.2564 | 1.0000 |
| Right_DefaultC_PHC_1 | -0.6167 | 1.0000 |
| Right_DefaultC_PHC_2 | 0.8624 | 1.0000 |
| Right_TempPar_1 | 0.2412 | 1.0000 |
| Right_TempPar_2 | -2.0876 | 1.0000 |
| Right_TempPar_3 | -4.0641 | 0.0291 |
| Right_TempPar_4 | -1.6100 | 1.0000 |
| Right_TempPar_5 | -3.2809 | 0.4996 |
| Right_TempPar_6 | -0.0691 | 1.0000 |
| Right_TempPar_7 | 0.6597 | 1.0000 |
| Right_TempPar_8 | -4.5743 | 0.0036 |
| Right_TempPar_9 | -1.2567 | 1.0000 |
| Right_TempPar_10 | 1.5002 | 1.0000 |

Table S6.Independent Samples t-Test Differences in Global Parameters between OCD and HC Groups.

| Global Parameters | t | P |
| --- | --- | --- |
| aCp | 1.350 | 0.894 |
| aGamma | -0.905 | 1 |
| aLambda | 2.749 | 0.034* |
| aLp | 2.106 | 0.183 |
| aSigma | -1.114 | 1 |
| aEg | -2.200 | 0.029* |
| aEloc | 1.295 | 0.197 |

Abbreviations:Cp:Clustering Coefficient;Gamma:Normalized Clustering Coefficient;Lambda:Normalized Path Length;Lp :Shortest Path Length;Sigma:Small-Worldness Sigma *p<0.05

Table S7.Independent Samples t-Test Differences in Node Parameters between OCD and HC Groups.

|  | T (DC) | P | T(Ne) | P |
| --- | --- | --- | --- | --- |
| Left_VisCent_ExStr_1 | 0.6290 | 1.0000 | 0.4744 | 1.0000 |
| Left_VisCent_ExStr_2 | 2.0020 | 1.0000 | 1.7589 | 1.0000 |
| Left_VisCent_ExStr_3 | -0.1938 | 1.0000 | -0.5082 | 1.0000 |
| Left_VisCent_ExStr_4 | -0.5437 | 1.0000 | -1.0059 | 1.0000 |
| Left_VisCent_ExStr_5 | 0.9661 | 1.0000 | 0.5248 | 1.0000 |
| Left_VisCent_ExStr_6 | 0.1015 | 1.0000 | -0.1740 | 1.0000 |
| Left_VisCent_ExStr_7 | 1.1944 | 1.0000 | 0.7743 | 1.0000 |
| Left_VisCent_ExStr_8 | -0.2081 | 1.0000 | -0.4776 | 1.0000 |
| Left_VisCent_ExStr_9 | 0.3012 | 1.0000 | 0.0529 | 1.0000 |
| Left_VisCent_ExStr_10 | 0.3844 | 1.0000 | -0.1512 | 1.0000 |
| Left_VisCent_ExStr_11 | -0.4057 | 1.0000 | -0.7301 | 1.0000 |
| Left_VisCent_ExStr_12 | 1.5244 | 1.0000 | 1.2779 | 1.0000 |
| Left_VisPeri_ExStrSup_1 | 1.0666 | 1.0000 | 0.5857 | 1.0000 |
| Left_VisPeri_ExStrSup_2 | 0.2381 | 1.0000 | -0.2068 | 1.0000 |
| Left_VisPeri_ExStrSup_3 | 1.0970 | 1.0000 | 0.6872 | 1.0000 |
| Left_VisPeri_ExStrSup_4 | -1.2074 | 1.0000 | -1.6559 | 1.0000 |
| Left_VisPeri_ExStrSup_5 | -0.1796 | 1.0000 | -0.4051 | 1.0000 |
| Left_VisPeri_ExStrSup_6 | -1.2111 | 1.0000 | -1.6933 | 1.0000 |
| Left_VisPeri_ExStrSup_7 | 0.2375 | 1.0000 | -0.4888 | 1.0000 |
| Left_VisPeri_ExStrSup_8 | -0.3780 | 1.0000 | -0.6912 | 1.0000 |
| Left_VisPeri_ExStrSup_9 | -1.4887 | 1.0000 | -1.9998 | 1.0000 |
| Left_VisPeri_ExStrSup_10 | -1.2950 | 1.0000 | -1.9052 | 1.0000 |
| Left_VisPeri_ExStrSup_11 | -1.6007 | 1.0000 | -1.9718 | 1.0000 |
| Left_VisPeri_ExStrSup_12 | 0.5246 | 1.0000 | -0.1991 | 1.0000 |
| Left_SomMotA_1 | -1.5772 | 1.0000 | -2.0781 | 1.0000 |
| Left_SomMotA_2 | -2.7147 | 1.0000 | -2.9854 | 1.0000 |
| Left_SomMotA_3 | -0.3580 | 1.0000 | -0.9024 | 1.0000 |
| Left_SomMotA_4 | 0.3263 | 1.0000 | -0.2594 | 1.0000 |
| Left_SomMotA_5 | -0.1413 | 1.0000 | -0.7172 | 1.0000 |
| Left_SomMotA_6 | -2.2467 | 1.0000 | -2.6086 | 1.0000 |
| Left_SomMotA_7 | -1.2255 | 1.0000 | -1.5338 | 1.0000 |
| Left_SomMotA_8 | -0.0507 | 1.0000 | -0.2476 | 1.0000 |
| Left_SomMotA_9 | 0.5717 | 1.0000 | 0.3863 | 1.0000 |
| Left_SomMotA_10 | -0.0538 | 1.0000 | -0.1809 | 1.0000 |
| Left_SomMotA_11 | 1.3139 | 1.0000 | 0.9423 | 1.0000 |
| Left_SomMotA_12 | 1.4575 | 1.0000 | 1.1195 | 1.0000 |
| Left_SomMotA_13 | -1.3000 | 1.0000 | -1.6022 | 1.0000 |
| Left_SomMotA_14 | 0.4848 | 1.0000 | 0.1211 | 1.0000 |
| Left_SomMotA_15 | 1.8380 | 1.0000 | 1.7823 | 1.0000 |
| Left_SomMotA_16 | 1.7432 | 1.0000 | 1.4524 | 1.0000 |
| Left_SomMotA_17 | 0.1808 | 1.0000 | -0.0787 | 1.0000 |
| Left_SomMotA_18 | 2.1465 | 1.0000 | 1.8791 | 1.0000 |
| Left_SomMotA_19 | 1.5695 | 1.0000 | 1.3231 | 1.0000 |
| Left_SomMotB_Aud_1 | 0.4023 | 1.0000 | 0.4628 | 1.0000 |
| Left_SomMotB_Aud_2 | 0.2613 | 1.0000 | -0.0273 | 1.0000 |
| Left_SomMotB_Aud_3 | 1.0327 | 1.0000 | 0.6677 | 1.0000 |
| Left_SomMotB_Aud_4 | 2.1019 | 1.0000 | 1.8565 | 1.0000 |
| Left_SomMotB_Aud_5 | 1.4346 | 1.0000 | 1.2530 | 1.0000 |
| Left_SomMotB_Aud_6 | 0.5780 | 1.0000 | 0.3445 | 1.0000 |
| Left_SomMotB_Aud_7 | 0.9205 | 1.0000 | 0.7087 | 1.0000 |
| Left_SomMotB_Aud_8 | 1.8542 | 1.0000 | 1.4103 | 1.0000 |
| Left_SomMotB_Aud_9 | 2.8339 | 1.0000 | 2.5795 | 1.0000 |
| Left_SomMotB_Aud_10 | 2.0064 | 1.0000 | 1.4390 | 1.0000 |
| Left_SomMotB_Aud_11 | 1.5181 | 1.0000 | 1.3133 | 1.0000 |
| Left_SomMotB_Aud_12 | 1.1442 | 1.0000 | 0.8444 | 1.0000 |
| Left_SomMotB_Aud_13 | 0.7406 | 1.0000 | 0.5231 | 1.0000 |
| Left_SomMotB_Aud_14 | 1.5895 | 1.0000 | 1.1642 | 1.0000 |
| Left_SomMotB_Aud_15 | 0.7767 | 1.0000 | 0.0427 | 1.0000 |
| Left_SomMotB_Aud_16 | 0.1195 | 1.0000 | -0.3130 | 1.0000 |
| Left_DorsAttnA_TempOcc_1 | 1.4157 | 1.0000 | 0.9959 | 1.0000 |
| Left_DorsAttnA_TempOcc_2 | 1.7025 | 1.0000 | 1.4319 | 1.0000 |
| Left_DorsAttnA_TempOcc_3 | 1.6210 | 1.0000 | 1.4241 | 1.0000 |
| Left_DorsAttnA_TempOcc_4 | 1.1823 | 1.0000 | 1.0500 | 1.0000 |
| Left_DorsAttnA_ParOcc_1 | 2.3254 | 1.0000 | 1.8914 | 1.0000 |
| Left_DorsAttnA_SPL_1 | 1.7798 | 1.0000 | 1.7596 | 1.0000 |
| Left_DorsAttnA_SPL_2 | 0.8363 | 1.0000 | 0.5522 | 1.0000 |
| Left_DorsAttnA_SPL_3 | 0.8933 | 1.0000 | 0.5192 | 1.0000 |
| Left_DorsAttnA_SPL_4 | 1.7990 | 1.0000 | 1.0510 | 1.0000 |
| Left_DorsAttnA_SPL_5 | 0.4450 | 1.0000 | 0.1350 | 1.0000 |
| Left_DorsAttnA_SPL_6 | -0.1622 | 1.0000 | -0.3888 | 1.0000 |
| Left_DorsAttnA_SPL_7 | 1.1174 | 1.0000 | 1.2077 | 1.0000 |
| Left_DorsAttnA_SPL_8 | -0.9142 | 1.0000 | -1.1447 | 1.0000 |
| Left_DorsAttnB_PostC_1 | -1.0200 | 1.0000 | -1.4807 | 1.0000 |
| Left_DorsAttnB_PostC_2 | 2.3701 | 1.0000 | 2.0732 | 1.0000 |
| Left_DorsAttnB_PostC_3 | -1.0754 | 1.0000 | -1.5059 | 1.0000 |
| Left_DorsAttnB_PostC_4 | 1.6947 | 1.0000 | 1.4189 | 1.0000 |
| Left_DorsAttnB_PostC_5 | -0.4038 | 1.0000 | -0.5143 | 1.0000 |
| Left_DorsAttnB_PostC_6 | -0.0287 | 1.0000 | -0.3315 | 1.0000 |
| Left_DorsAttnB_PostC_7 | 1.1078 | 1.0000 | 0.7964 | 1.0000 |
| Left_DorsAttnB_PostC_8 | -0.4580 | 1.0000 | -0.0750 | 1.0000 |
| Left_DorsAttnB_PostC_9 | -1.8378 | 1.0000 | -2.0618 | 1.0000 |
| Left_DorsAttnB_FEF_1 | 0.2480 | 1.0000 | -0.0615 | 1.0000 |
| Left_DorsAttnB_FEF_2 | -0.7874 | 1.0000 | -1.0221 | 1.0000 |
| Left_DorsAttnB_FEF_3 | -0.5168 | 1.0000 | -0.1851 | 1.0000 |
| Left_DorsAttnB_PrCv_1 | 0.7600 | 1.0000 | 0.5214 | 1.0000 |
| Left_SalVentAttnA_ParOper_1 | -0.2365 | 1.0000 | -0.4630 | 1.0000 |
| Left_SalVentAttnA_ParOper_2 | -0.0393 | 1.0000 | -0.3520 | 1.0000 |
| Left_SalVentAttnA_ParOper_3 | 0.0651 | 1.0000 | -0.2735 | 1.0000 |
| Left_SalVentAttnA_Ins_1 | -0.2410 | 1.0000 | -0.9892 | 1.0000 |
| Left_SalVentAttnA_Ins_2 | 0.6153 | 1.0000 | 0.3015 | 1.0000 |
| Left_SalVentAttnA_Ins_3 | 0.4746 | 1.0000 | 0.2488 | 1.0000 |
| Left_SalVentAttnA_Ins_4 | -1.1065 | 1.0000 | -1.3433 | 1.0000 |
| Left_SalVentAttnA_Ins_5 | -2.6797 | 1.0000 | -2.8100 | 1.0000 |
| Left_SalVentAttnA_Ins_6 | 1.2464 | 1.0000 | 0.7056 | 1.0000 |
| Left_SalVentAttnA_ParMed_1 | -1.5695 | 1.0000 | -1.8348 | 1.0000 |
| Left_SalVentAttnA_ParMed_2 | 1.9735 | 1.0000 | 1.5756 | 1.0000 |
| Left_SalVentAttnA_ParMed_3 | 0.2528 | 1.0000 | -0.6243 | 1.0000 |
| Left_SalVentAttnA_FrMed_1 | -1.4573 | 1.0000 | -1.6419 | 1.0000 |
| Left_SalVentAttnA_FrMed_2 | -1.7016 | 1.0000 | -2.1716 | 1.0000 |
| Left_SalVentAttnA_FrMed_3 | -0.9604 | 1.0000 | -1.0052 | 1.0000 |
| Left_SalVentAttnB_PFCl_1 | -1.0906 | 1.0000 | -1.3929 | 1.0000 |
| Left_SalVentAttnB_PFCl_2 | -1.1171 | 1.0000 | -1.5852 | 1.0000 |
| Left_SalVentAttnB_PFCl_3 | -1.5210 | 1.0000 | -1.6761 | 1.0000 |
| Left_SalVentAttnB_PFCv_1 | -0.1762 | 1.0000 | -0.3151 | 1.0000 |
| Left_SalVentAttnB_PFCv_2 | -0.9253 | 1.0000 | -1.2776 | 1.0000 |
| Left_SalVentAttnB_PFCv_3 | -2.9575 | 1.0000 | -3.2048 | 0.6426 |
| Left_SalVentAttnB_OFC_1 | -2.3470 | 1.0000 | -2.6327 | 1.0000 |
| Left_SalVentAttnB_PFCmp_1 | -1.8477 | 1.0000 | -1.9382 | 1.0000 |
| Left_Limbic_OFC_1 | 0.2054 | 1.0000 | -0.0909 | 1.0000 |
| Left_Limbic_OFC_2 | -2.3194 | 1.0000 | -2.5330 | 1.0000 |
| Left_Limbic_OFC_3 | -1.2841 | 1.0000 | -1.5048 | 1.0000 |
| Left_Limbic_OFC_4 | -1.6708 | 1.0000 | -1.8206 | 1.0000 |
| Left_Limbic_OFC_5 | 0.2189 | 1.0000 | 0.0473 | 1.0000 |
| Left_Limbic_TempPole_1 | 1.8681 | 1.0000 | 1.1221 | 1.0000 |
| Left_Limbic_TempPole_2 | 1.1439 | 1.0000 | 0.9444 | 1.0000 |
| Left_Limbic_TempPole_3 | 1.9204 | 1.0000 | 1.5339 | 1.0000 |
| Left_Limbic_TempPole_4 | 1.9607 | 1.0000 | 1.3386 | 1.0000 |
| Left_Limbic_TempPole_5 | 0.5278 | 1.0000 | 0.1980 | 1.0000 |
| Left_Limbic_TempPole_6 | 1.7802 | 1.0000 | 1.4363 | 1.0000 |
| Left_Limbic_TempPole_7 | 3.1712 | 0.7171 | 2.2568 | 1.0000 |
| Left_ContA_Temp_1 | 2.6195 | 1.0000 | 2.3558 | 1.0000 |
| Left_ContA_IPS_1 | 2.1259 | 1.0000 | 1.8040 | 1.0000 |
| Left_ContA_IPS_2 | 1.4959 | 1.0000 | 1.3722 | 1.0000 |
| Left_ContA_IPS_3 | 3.5295 | 0.2129 | 2.8688 | 1.0000 |
| Left_ContA_IPS_4 | 1.5548 | 1.0000 | 0.6518 | 1.0000 |
| Left_ContA_IPS_5 | 2.7988 | 1.0000 | 1.6833 | 1.0000 |
| Left_ContA_PFCd_1 | -1.7619 | 1.0000 | -2.1504 | 1.0000 |
| Left_ContA_PFCl_1 | -0.3781 | 1.0000 | -0.4299 | 1.0000 |
| Left_ContA_PFCl_2 | -1.8355 | 1.0000 | -1.5152 | 1.0000 |
| Left_ContA_PFCl_3 | -1.6918 | 1.0000 | -2.0963 | 1.0000 |
| Left_ContA_PFCl_4 | -2.0965 | 1.0000 | -1.5445 | 1.0000 |
| Left_ContA_PFCl_5 | 0.3959 | 1.0000 | 0.5749 | 1.0000 |
| Left_ContA_Cinga_1 | -1.1379 | 1.0000 | -1.3828 | 1.0000 |
| Left_ContB_Temp_1 | -1.4743 | 1.0000 | -1.9394 | 1.0000 |
| Left_ContB_Temp_2 | -2.0193 | 1.0000 | -2.4809 | 1.0000 |
| Left_ContB_IPL_1 | -0.8875 | 1.0000 | -1.4346 | 1.0000 |
| Left_ContB_IPL_2 | -3.1006 | 0.9005 | -3.5476 | 0.1996 |
| Left_ContB_IPL_3 | -0.9704 | 1.0000 | -1.4361 | 1.0000 |
| Left_ContB_PFCd_1 | -2.1338 | 1.0000 | -2.6621 | 1.0000 |
| Left_ContB_PFClv_1 | 0.8345 | 1.0000 | 0.3545 | 1.0000 |
| Left_ContB_PFClv_2 | -0.3961 | 1.0000 | -0.8682 | 1.0000 |
| Left_ContB_PFClv_3 | -1.8210 | 1.0000 | -2.0497 | 1.0000 |
| Left_ContB_PFCmp_1 | -0.8309 | 1.0000 | -1.0936 | 1.0000 |
| Left_ContC_pCun_1 | -2.2917 | 1.0000 | -2.6639 | 1.0000 |
| Left_ContC_pCun_2 | -2.5678 | 1.0000 | -2.7197 | 1.0000 |
| Left_ContC_pCun_3 | -1.7345 | 1.0000 | -1.8301 | 1.0000 |
| Left_ContC_Cingp_1 | -1.6166 | 1.0000 | -2.0722 | 1.0000 |
| Left_ContC_Cingp_2 | 0.3171 | 1.0000 | -0.0716 | 1.0000 |
| Left_DefaultA_IPL_1 | 2.2337 | 1.0000 | 1.9281 | 1.0000 |
| Left_DefaultA_IPL_2 | -0.9528 | 1.0000 | -1.1848 | 1.0000 |
| Left_DefaultA_PFCd_1 | 0.3118 | 1.0000 | 0.2492 | 1.0000 |
| Left_DefaultA_PFCd_2 | 1.0462 | 1.0000 | 0.8163 | 1.0000 |
| Left_DefaultA_PFCd_3 | 0.8417 | 1.0000 | 0.6720 | 1.0000 |
| Left_DefaultA_PCC_1 | 0.0051 | 1.0000 | -0.1827 | 1.0000 |
| Left_DefaultA_PCC_2 | 0.6375 | 1.0000 | 0.7756 | 1.0000 |
| Left_DefaultA_PCC_3 | 0.2608 | 1.0000 | 0.1980 | 1.0000 |
| Left_DefaultA_PCC_4 | -0.7072 | 1.0000 | -0.9287 | 1.0000 |
| Left_DefaultA_PCC_5 | 0.9072 | 1.0000 | 0.6675 | 1.0000 |
| Left_DefaultA_PCC_6 | 2.0042 | 1.0000 | 1.6582 | 1.0000 |
| Left_DefaultA_PCC_7 | -0.6765 | 1.0000 | -1.0288 | 1.0000 |
| Left_DefaultA_PFCm_1 | -0.3637 | 1.0000 | -0.8194 | 1.0000 |
| Left_DefaultA_PFCm_2 | -1.3865 | 1.0000 | -1.8333 | 1.0000 |
| Left_DefaultA_PFCm_3 | -1.9533 | 1.0000 | -2.3518 | 1.0000 |
| Left_DefaultA_PFCm_4 | -1.0047 | 1.0000 | -1.3042 | 1.0000 |
| Left_DefaultA_PFCm_5 | -2.5159 | 1.0000 | -1.9020 | 1.0000 |
| Left_DefaultA_PFCm_6 | -1.8618 | 1.0000 | -2.0095 | 1.0000 |
| Left_DefaultB_Temp_1 | -2.2293 | 1.0000 | -2.5821 | 1.0000 |
| Left_DefaultB_Temp_2 | 0.7759 | 1.0000 | 0.3169 | 1.0000 |
| Left_DefaultB_Temp_3 | 1.3638 | 1.0000 | 0.6415 | 1.0000 |
| Left_DefaultB_Temp_4 | -0.9336 | 1.0000 | -1.3097 | 1.0000 |
| Left_DefaultB_Temp_5 | -1.4946 | 1.0000 | -2.0276 | 1.0000 |
| Left_DefaultB_Temp_6 | 0.1413 | 1.0000 | -0.4021 | 1.0000 |
| Left_DefaultB_IPL_1 | 0.0457 | 1.0000 | -0.3638 | 1.0000 |
| Left_DefaultB_IPL_2 | -0.4139 | 1.0000 | -0.9306 | 1.0000 |
| Left_DefaultB_PFCd_1 | -0.0487 | 1.0000 | -0.5523 | 1.0000 |
| Left_DefaultB_PFCd_2 | 0.3621 | 1.0000 | 0.2992 | 1.0000 |
| Left_DefaultB_PFCd_3 | -1.6255 | 1.0000 | -2.0265 | 1.0000 |
| Left_DefaultB_PFCd_4 | -0.3011 | 1.0000 | -0.7204 | 1.0000 |
| Left_DefaultB_PFCd_5 | -0.1640 | 1.0000 | -0.6260 | 1.0000 |
| Left_DefaultB_PFCd_6 | -1.0180 | 1.0000 | -1.4679 | 1.0000 |
| Left_DefaultB_PFCl_1 | -1.1899 | 1.0000 | -1.5525 | 1.0000 |
| Left_DefaultB_PFCl_2 | -0.0884 | 1.0000 | -0.8360 | 1.0000 |
| Left_DefaultB_PFCv_1 | -0.2005 | 1.0000 | -1.0871 | 1.0000 |
| Left_DefaultB_PFCv_2 | -0.5950 | 1.0000 | -1.1008 | 1.0000 |
| Left_DefaultB_PFCv_3 | -0.2401 | 1.0000 | -0.8316 | 1.0000 |
| Left_DefaultB_PFCv_4 | -0.7670 | 1.0000 | -1.2748 | 1.0000 |
| Left_DefaultB_PFCv_5 | -1.2987 | 1.0000 | -1.8123 | 1.0000 |
| Left_DefaultC_IPL_1 | 0.4012 | 1.0000 | -0.9372 | 1.0000 |
| Left_DefaultC_Rsp_1 | 1.0530 | 1.0000 | 0.5220 | 1.0000 |
| Left_DefaultC_Rsp_2 | -0.0607 | 1.0000 | -0.3597 | 1.0000 |
| Left_DefaultC_Rsp_3 | -0.9785 | 1.0000 | -1.4465 | 1.0000 |
| Left_DefaultC_PHC_1 | -0.5550 | 1.0000 | -0.9888 | 1.0000 |
| Left_DefaultC_PHC_2 | -0.5273 | 1.0000 | -0.7473 | 1.0000 |
| Left_DefaultC_PHC_3 | -3.0419 | 1.0000 | -3.2868 | 0.4898 |
| Left_TempPar_1 | -1.1508 | 1.0000 | -1.6192 | 1.0000 |
| Left_TempPar_2 | -1.0792 | 1.0000 | -1.5356 | 1.0000 |
| Left_TempPar_3 | -1.6816 | 1.0000 | -1.9525 | 1.0000 |
| Left_TempPar_4 | -1.3220 | 1.0000 | -1.5076 | 1.0000 |
| Left_TempPar_5 | -1.6673 | 1.0000 | -2.2749 | 1.0000 |
| Left_TempPar_6 | -0.8571 | 1.0000 | -1.1554 | 1.0000 |
| Right_VisCent_ExStr_1 | 1.7790 | 1.0000 | 1.4269 | 1.0000 |
| Right_VisCent_ExStr_2 | 1.4532 | 1.0000 | 1.0827 | 1.0000 |
| Right_VisCent_ExStr_3 | 0.9019 | 1.0000 | 0.2661 | 1.0000 |
| Right_VisCent_ExStr_4 | -1.0446 | 1.0000 | -1.3269 | 1.0000 |
| Right_VisCent_ExStr_5 | 0.9929 | 1.0000 | 0.3266 | 1.0000 |
| Right_VisCent_ExStr_6 | 0.0710 | 1.0000 | -0.4147 | 1.0000 |
| Right_VisCent_ExStr_7 | 0.6423 | 1.0000 | 0.3180 | 1.0000 |
| Right_VisCent_ExStr_8 | 0.3267 | 1.0000 | 0.2804 | 1.0000 |
| Right_VisCent_ExStr_9 | -0.9364 | 1.0000 | -1.3289 | 1.0000 |
| Right_VisCent_ExStr_10 | -0.3545 | 1.0000 | -0.6624 | 1.0000 |
| Right_VisCent_ExStr_11 | -0.7166 | 1.0000 | -1.1152 | 1.0000 |
| Right_VisCent_ExStr_12 | -0.3662 | 1.0000 | -0.8863 | 1.0000 |
| Right_VisPeri_ExStrSup_1 | 0.5288 | 1.0000 | 0.3006 | 1.0000 |
| Right_VisPeri_ExStrSup_2 | 2.0305 | 1.0000 | 1.7033 | 1.0000 |
| Right_VisPeri_ExStrSup_3 | -0.3511 | 1.0000 | -0.4894 | 1.0000 |
| Right_VisPeri_ExStrSup_4 | 0.1892 | 1.0000 | -0.1866 | 1.0000 |
| Right_VisPeri_ExStrSup_5 | 0.6537 | 1.0000 | 0.3047 | 1.0000 |
| Right_VisPeri_ExStrSup_6 | 1.9077 | 1.0000 | 0.9773 | 1.0000 |
| Right_VisPeri_ExStrSup_7 | -1.3758 | 1.0000 | -1.9543 | 1.0000 |
| Right_VisPeri_ExStrSup_8 | -0.0147 | 1.0000 | -0.7020 | 1.0000 |
| Right_VisPeri_ExStrSup_9 | 1.5868 | 1.0000 | 0.9423 | 1.0000 |
| Right_VisPeri_ExStrSup_10 | -0.3238 | 1.0000 | -0.6529 | 1.0000 |
| Right_VisPeri_ExStrSup_11 | 0.4692 | 1.0000 | -0.3852 | 1.0000 |
| Right_SomMotA_1 | -1.8830 | 1.0000 | -2.2367 | 1.0000 |
| Right_SomMotA_2 | -1.8462 | 1.0000 | -2.3850 | 1.0000 |
| Right_SomMotA_3 | 0.6881 | 1.0000 | -0.1416 | 1.0000 |
| Right_SomMotA_4 | -0.4680 | 1.0000 | -0.7831 | 1.0000 |
| Right_SomMotA_5 | -2.1104 | 1.0000 | -2.5605 | 1.0000 |
| Right_SomMotA_6 | -0.6176 | 1.0000 | -1.4065 | 1.0000 |
| Right_SomMotA_7 | -0.7064 | 1.0000 | -0.9978 | 1.0000 |
| Right_SomMotA_8 | -0.9455 | 1.0000 | -1.1477 | 1.0000 |
| Right_SomMotA_9 | 1.0816 | 1.0000 | 0.8160 | 1.0000 |
| Right_SomMotA_10 | -1.2711 | 1.0000 | -1.4821 | 1.0000 |
| Right_SomMotA_11 | -0.6807 | 1.0000 | -1.0047 | 1.0000 |
| Right_SomMotA_12 | 2.2116 | 1.0000 | 2.2313 | 1.0000 |
| Right_SomMotA_13 | 0.3532 | 1.0000 | 0.3181 | 1.0000 |
| Right_SomMotA_14 | -0.5538 | 1.0000 | -0.7839 | 1.0000 |
| Right_SomMotA_15 | 0.3351 | 1.0000 | -0.1883 | 1.0000 |
| Right_SomMotA_16 | 1.1444 | 1.0000 | 1.0169 | 1.0000 |
| Right_SomMotA_17 | -1.0407 | 1.0000 | -1.1143 | 1.0000 |
| Right_SomMotA_18 | 0.7242 | 1.0000 | 0.5993 | 1.0000 |
| Right_SomMotA_19 | 1.0762 | 1.0000 | 0.8314 | 1.0000 |
| Right_SomMotA_20 | -0.2990 | 1.0000 | -0.4801 | 1.0000 |
| Right_SomMotB_S2_1 | 1.2576 | 1.0000 | 1.0826 | 1.0000 |
| Right_SomMotB_S2_2 | 0.4082 | 1.0000 | 0.8623 | 1.0000 |
| Right_SomMotB_S2_3 | 0.6072 | 1.0000 | 0.4878 | 1.0000 |
| Right_SomMotB_S2_4 | -0.4172 | 1.0000 | -0.7554 | 1.0000 |
| Right_SomMotB_S2_5 | 0.4603 | 1.0000 | 0.2549 | 1.0000 |
| Right_SomMotB_S2_6 | 0.5073 | 1.0000 | 0.2377 | 1.0000 |
| Right_SomMotB_S2_7 | -0.7648 | 1.0000 | -1.1016 | 1.0000 |
| Right_SomMotB_S2_8 | 0.6524 | 1.0000 | 0.2163 | 1.0000 |
| Right_SomMotB_S2_9 | -0.1068 | 1.0000 | -0.4521 | 1.0000 |
| Right_SomMotB_S2_10 | 0.7667 | 1.0000 | 0.5517 | 1.0000 |
| Right_SomMotB_S2_11 | -0.1753 | 1.0000 | -0.3987 | 1.0000 |
| Right_SomMotB_S2_12 | 0.7226 | 1.0000 | 0.2224 | 1.0000 |
| Right_SomMotB_S2_13 | -0.4252 | 1.0000 | -0.9829 | 1.0000 |
| Right_SomMotB_S2_14 | 1.1177 | 1.0000 | 0.7783 | 1.0000 |
| Right_SomMotB_S2_15 | 0.7119 | 1.0000 | 0.5583 | 1.0000 |
| Right_DorsAttnA_TempOcc_1 | 0.2756 | 1.0000 | -0.0917 | 1.0000 |
| Right_DorsAttnA_TempOcc_2 | 0.8253 | 1.0000 | 0.4692 | 1.0000 |
| Right_DorsAttnA_TempOcc_3 | 0.5668 | 1.0000 | -0.1321 | 1.0000 |
| Right_DorsAttnA_TempOcc_4 | 1.7599 | 1.0000 | 1.1180 | 1.0000 |
| Right_DorsAttnA_TempOcc_5 | 0.0617 | 1.0000 | -0.1721 | 1.0000 |
| Right_DorsAttnA_SPL_1 | 1.1250 | 1.0000 | 0.9243 | 1.0000 |
| Right_DorsAttnA_SPL_2 | 0.5449 | 1.0000 | 0.0165 | 1.0000 |
| Right_DorsAttnA_SPL_3 | 0.0073 | 1.0000 | -0.0315 | 1.0000 |
| Right_DorsAttnA_SPL_4 | 1.1216 | 1.0000 | 0.3176 | 1.0000 |
| Right_DorsAttnA_SPL_5 | 2.1461 | 1.0000 | 1.7333 | 1.0000 |
| Right_DorsAttnA_SPL_6 | -0.0620 | 1.0000 | -0.3081 | 1.0000 |
| Right_DorsAttnA_SPL_7 | 1.6996 | 1.0000 | 0.9568 | 1.0000 |
| Right_DorsAttnA_SPL_8 | -1.5432 | 1.0000 | -1.9671 | 1.0000 |
| Right_DorsAttnA_SPL_9 | -0.1208 | 1.0000 | -0.3422 | 1.0000 |
| Right_DorsAttnB_TempOcc_1 | 0.4200 | 1.0000 | 0.1277 | 1.0000 |
| Right_DorsAttnB_PostC_1 | -0.7116 | 1.0000 | -1.2659 | 1.0000 |
| Right_DorsAttnB_PostC_2 | -1.0088 | 1.0000 | -1.4621 | 1.0000 |
| Right_DorsAttnB_PostC_3 | 1.5387 | 1.0000 | 1.1842 | 1.0000 |
| Right_DorsAttnB_PostC_4 | -0.8910 | 1.0000 | -1.5073 | 1.0000 |
| Right_DorsAttnB_PostC_5 | -0.3816 | 1.0000 | -0.8127 | 1.0000 |
| Right_DorsAttnB_PostC_6 | 0.4299 | 1.0000 | -0.0008 | 1.0000 |
| Right_DorsAttnB_PostC_7 | -0.5390 | 1.0000 | -1.1757 | 1.0000 |
| Right_DorsAttnB_PostC_8 | -2.1035 | 1.0000 | -2.4897 | 1.0000 |
| Right_DorsAttnB_FEF_1 | -0.7387 | 1.0000 | -1.3879 | 1.0000 |
| Right_DorsAttnB_FEF_2 | -0.4370 | 1.0000 | -0.8313 | 1.0000 |
| Right_DorsAttnB_FEF_3 | -0.4808 | 1.0000 | -0.9872 | 1.0000 |
| Right_SalVentAttnA_ParOper_1 | -0.8788 | 1.0000 | -1.3618 | 1.0000 |
| Right_SalVentAttnA_ParOper_2 | -1.3724 | 1.0000 | -1.5486 | 1.0000 |
| Right_SalVentAttnA_ParOper_3 | -0.6857 | 1.0000 | -1.0087 | 1.0000 |
| Right_SalVentAttnA_PrC_1 | -0.1819 | 1.0000 | -0.8282 | 1.0000 |
| Right_SalVentAttnA_Ins_1 | 0.4371 | 1.0000 | -0.5430 | 1.0000 |
| Right_SalVentAttnA_Ins_2 | 0.0371 | 1.0000 | -0.1767 | 1.0000 |
| Right_SalVentAttnA_Ins_3 | 0.2720 | 1.0000 | -0.1149 | 1.0000 |
| Right_SalVentAttnA_Ins_4 | 1.1447 | 1.0000 | 1.3909 | 1.0000 |
| Right_SalVentAttnA_Ins_5 | -0.2273 | 1.0000 | -0.4182 | 1.0000 |
| Right_SalVentAttnA_Ins_6 | 2.4355 | 1.0000 | 2.2561 | 1.0000 |
| Right_SalVentAttnA_Ins_7 | 0.9780 | 1.0000 | 0.7184 | 1.0000 |
| Right_SalVentAttnA_ParMed_1 | -0.5839 | 1.0000 | -1.0607 | 1.0000 |
| Right_SalVentAttnA_ParMed_2 | -1.6805 | 1.0000 | -1.9592 | 1.0000 |
| Right_SalVentAttnA_ParMed_3 | -1.3779 | 1.0000 | -1.6824 | 1.0000 |
| Right_SalVentAttnA_ParMed_4 | 0.3977 | 1.0000 | 0.1753 | 1.0000 |
| Right_SalVentAttnA_ParMed_5 | -0.6711 | 1.0000 | -1.2599 | 1.0000 |
| Right_SalVentAttnA_ParMed_6 | 0.3392 | 1.0000 | 0.1201 | 1.0000 |
| Right_SalVentAttnA_ParMed_7 | -0.2837 | 1.0000 | -0.6484 | 1.0000 |
| Right_SalVentAttnA_ParMed_8 | -1.2684 | 1.0000 | -1.3874 | 1.0000 |
| Right_SalVentAttnB_IPL_1 | 0.5854 | 1.0000 | 0.2072 | 1.0000 |
| Right_SalVentAttnB_PFCl_1 | 0.0946 | 1.0000 | -0.2344 | 1.0000 |
| Right_SalVentAttnB_PFCl_2 | -1.6231 | 1.0000 | -1.9544 | 1.0000 |
| Right_SalVentAttnB_PFCl_3 | -1.6182 | 1.0000 | -1.6708 | 1.0000 |
| Right_SalVentAttnB_PFCl_4 | -0.8965 | 1.0000 | -0.9333 | 1.0000 |
| Right_SalVentAttnB_PFCv_1 | -0.9530 | 1.0000 | -1.1200 | 1.0000 |
| Right_SalVentAttnB_PFCv_2 | -3.0561 | 1.0000 | -3.3444 | 0.4035 |
| Right_SalVentAttnB_PFCmp_1 | -2.8565 | 1.0000 | -3.0137 | 1.0000 |
| Right_SalVentAttnB_PFCmp_2 | -1.4813 | 1.0000 | -1.6337 | 1.0000 |
| Right_Limbic_OFC_1 | -1.7249 | 1.0000 | -2.0035 | 1.0000 |
| Right_Limbic_OFC_2 | -0.2479 | 1.0000 | -0.2846 | 1.0000 |
| Right_Limbic_OFC_3 | -0.1614 | 1.0000 | -0.4158 | 1.0000 |
| Right_Limbic_OFC_4 | 0.1558 | 1.0000 | -0.1632 | 1.0000 |
| Right_Limbic_OFC_5 | 0.0525 | 1.0000 | -0.2734 | 1.0000 |
| Right_Limbic_OFC_6 | 0.5603 | 1.0000 | 0.2845 | 1.0000 |
| Right_Limbic_TempPole_1 | 1.5941 | 1.0000 | 1.2364 | 1.0000 |
| Right_Limbic_TempPole_2 | 2.6542 | 1.0000 | 2.3037 | 1.0000 |
| Right_Limbic_TempPole_3 | 1.5171 | 1.0000 | 1.2740 | 1.0000 |
| Right_Limbic_TempPole_4 | 1.5899 | 1.0000 | 1.1371 | 1.0000 |
| Right_Limbic_TempPole_5 | 2.3963 | 1.0000 | 1.6187 | 1.0000 |
| Right_Limbic_TempPole_6 | 1.0503 | 1.0000 | 0.6653 | 1.0000 |
| Right_ContA_IPS_1 | 1.7402 | 1.0000 | 1.8556 | 1.0000 |
| Right_ContA_IPS_2 | 2.3160 | 1.0000 | 2.0453 | 1.0000 |
| Right_ContA_IPS_3 | 2.5913 | 1.0000 | 2.2094 | 1.0000 |
| Right_ContA_IPS_4 | 2.9767 | 1.0000 | 2.2430 | 1.0000 |
| Right_ContA_PFCd_1 | 1.5022 | 1.0000 | 0.8302 | 1.0000 |
| Right_ContA_PFCl_1 | 1.5410 | 1.0000 | 0.6759 | 1.0000 |
| Right_ContA_PFCl_2 | 3.1453 | 0.7801 | 2.5282 | 1.0000 |
| Right_ContA_PFCl_3 | -1.2102 | 1.0000 | -1.8019 | 1.0000 |
| Right_ContA_PFCl_4 | -1.0758 | 1.0000 | -1.3283 | 1.0000 |
| Right_ContA_PFCl_5 | -0.9875 | 1.0000 | -1.3106 | 1.0000 |
| Right_ContA_Cinga_1 | 1.8744 | 1.0000 | 1.6111 | 1.0000 |
| Right_ContB_Temp_1 | -2.2294 | 1.0000 | -2.7553 | 1.0000 |
| Right_ContB_Temp_2 | -2.1716 | 1.0000 | -2.6318 | 1.0000 |
| Right_ContB_IPL_1 | 0.0348 | 1.0000 | -0.3539 | 1.0000 |
| Right_ContB_IPL_2 | -0.7474 | 1.0000 | -1.0977 | 1.0000 |
| Right_ContB_IPL_3 | -1.3671 | 1.0000 | -1.6355 | 1.0000 |
| Right_ContB_IPL_4 | -0.4923 | 1.0000 | -0.6362 | 1.0000 |
| Right_ContB_PFCld_1 | -2.5793 | 1.0000 | -2.9024 | 1.0000 |
| Right_ContB_PFCld_2 | -1.8171 | 1.0000 | -2.3752 | 1.0000 |
| Right_ContB_PFCld_3 | 0.2204 | 1.0000 | -0.2572 | 1.0000 |
| Right_ContB_PFCld_4 | -2.2094 | 1.0000 | -2.7615 | 1.0000 |
| Right_ContB_PFClv_1 | 0.5627 | 1.0000 | -0.0044 | 1.0000 |
| Right_ContB_PFClv_2 | 1.1667 | 1.0000 | 0.8876 | 1.0000 |
| Right_ContB_PFClv_3 | -2.2697 | 1.0000 | -2.6330 | 1.0000 |
| Right_ContB_PFClv_4 | -0.5177 | 1.0000 | -0.9744 | 1.0000 |
| Right_ContB_PFCmp_1 | -0.2724 | 1.0000 | -0.7570 | 1.0000 |
| Right_ContC_pCun_1 | -1.5336 | 1.0000 | -1.9500 | 1.0000 |
| Right_ContC_pCun_2 | 0.1526 | 1.0000 | -0.3296 | 1.0000 |
| Right_ContC_pCun_3 | 0.1980 | 1.0000 | 0.0011 | 1.0000 |
| Right_ContC_pCun_4 | -0.0949 | 1.0000 | -0.4166 | 1.0000 |
| Right_ContC_pCun_5 | 0.5228 | 1.0000 | -0.0129 | 1.0000 |
| Right_ContC_Cingp_1 | -3.6255 | 0.1512 | -3.8785 | 0.0594 |
| Right_ContC_Cingp_2 | -2.5216 | 1.0000 | -3.0007 | 1.0000 |
| Right_DefaultA_Temp_1 | -1.7807 | 1.0000 | -2.1599 | 1.0000 |
| Right_DefaultA_IPL_1 | -0.7177 | 1.0000 | -0.8140 | 1.0000 |
| Right_DefaultA_IPL_2 | -1.2641 | 1.0000 | -1.7178 | 1.0000 |
| Right_DefaultA_PFCd_1 | -0.0875 | 1.0000 | -0.3365 | 1.0000 |
| Right_DefaultA_PFCd_2 | -0.0484 | 1.0000 | -0.4536 | 1.0000 |
| Right_DefaultA_PCC_1 | -0.3567 | 1.0000 | -0.8102 | 1.0000 |
| Right_DefaultA_PCC_2 | -0.1957 | 1.0000 | -0.7546 | 1.0000 |
| Right_DefaultA_PCC_3 | -2.1730 | 1.0000 | -2.5277 | 1.0000 |
| Right_DefaultA_PCC_4 | -1.2445 | 1.0000 | -1.7534 | 1.0000 |
| Right_DefaultA_PCC_5 | 3.4127 | 0.3196 | 3.1589 | 0.7463 |
| Right_DefaultA_PFCm_1 | 0.6511 | 1.0000 | 0.2277 | 1.0000 |
| Right_DefaultA_PFCm_2 | 0.7800 | 1.0000 | 0.5309 | 1.0000 |
| Right_DefaultA_PFCm_3 | 0.9560 | 1.0000 | 0.6995 | 1.0000 |
| Right_DefaultA_PFCm_4 | -0.3584 | 1.0000 | -0.6867 | 1.0000 |
| Right_DefaultA_PFCm_5 | 2.2667 | 1.0000 | 2.1828 | 1.0000 |
| Right_DefaultA_PFCm_6 | 1.2586 | 1.0000 | 1.0324 | 1.0000 |
| Right_DefaultB_Temp_1 | 1.2380 | 1.0000 | 0.9057 | 1.0000 |
| Right_DefaultB_Temp_2 | -1.1709 | 1.0000 | -1.5970 | 1.0000 |
| Right_DefaultB_AntTemp_1 | -1.5671 | 1.0000 | -2.1199 | 1.0000 |
| Right_DefaultB_PFCd_1 | -1.0533 | 1.0000 | -1.3947 | 1.0000 |
| Right_DefaultB_PFCd_2 | 0.2641 | 1.0000 | -0.1151 | 1.0000 |
| Right_DefaultB_PFCd_3 | 1.1509 | 1.0000 | 0.5135 | 1.0000 |
| Right_DefaultB_PFCd_4 | 1.9557 | 1.0000 | 1.5800 | 1.0000 |
| Right_DefaultB_PFCd_5 | -0.1682 | 1.0000 | -0.6291 | 1.0000 |
| Right_DefaultB_PFCv_1 | 0.5899 | 1.0000 | 0.0300 | 1.0000 |
| Right_DefaultB_PFCv_2 | 0.4486 | 1.0000 | -0.2488 | 1.0000 |
| Right_DefaultB_PFCv_3 | -1.2578 | 1.0000 | -1.6933 | 1.0000 |
| Right_DefaultC_IPL_1 | -0.0885 | 1.0000 | -0.5184 | 1.0000 |
| Right_DefaultC_IPL_2 | -0.8461 | 1.0000 | -1.1658 | 1.0000 |
| Right_DefaultC_Rsp_1 | 1.3372 | 1.0000 | 0.6460 | 1.0000 |
| Right_DefaultC_Rsp_2 | -1.5258 | 1.0000 | -1.9396 | 1.0000 |
| Right_DefaultC_PHC_1 | -0.2772 | 1.0000 | -1.0772 | 1.0000 |
| Right_DefaultC_PHC_2 | -0.1026 | 1.0000 | -0.4492 | 1.0000 |
| Right_TempPar_1 | -0.2042 | 1.0000 | -0.6737 | 1.0000 |
| Right_TempPar_2 | -1.3715 | 1.0000 | -1.6834 | 1.0000 |
| Right_TempPar_3 | -0.0557 | 1.0000 | -0.4058 | 1.0000 |
| Right_TempPar_4 | -4.2827 | 0.0122 | -4.6866 | 0.0022 |
| Right_TempPar_5 | -0.7863 | 1.0000 | -1.2404 | 1.0000 |
| Right_TempPar_6 | -1.6519 | 1.0000 | -2.1769 | 1.0000 |
| Right_TempPar_7 | -1.6393 | 1.0000 | -1.9688 | 1.0000 |
| Right_TempPar_8 | -1.7559 | 1.0000 | -2.0642 | 1.0000 |
| Right_TempPar_9 | -1.1487 | 1.0000 | -1.5584 | 1.0000 |
| Right_TempPar_10 | -0.9532 | 1.0000 | -1.1800 | 1.0000 |

Table S8.Person correlation analysis between structural-functional coupling and clinical scores

|  | VIS | SMN | DAN | VAN | LIM | FPN | DMN | Left_VisPeri_ExStrSup_12 | Left_SomMotA_13 | Left_DorsAttnA_SPL_6 | Left_DefaultB_Temp_4 | Right_SomMotA_10 | Right_ContB_PFCld_1 | Right_TempPar_3 | Right_TempPar_8 |
| --- | --- | --- | --- | --- | --- | --- | --- | --- | --- | --- | --- | --- | --- | --- | --- |
| YBOCS | 0.027 | -0.004 | 0.03 | -0.197 | 0.059 | -0.03 | 0.079 | -0.095 | 0.113 | 0.158 | 0.068 | -0.093 | -0.066 | 0.218* | 0.19 |
| Obsession | 0.054 | 0.1 | 0.006 | -0.171 | 0.004 | -0.052 | 0.009 | -0.061 | 0.122 | 0.098 | 0.065 | -0.126 | -0.097 | 0.2 | 0.132 |
| Compulsion | -0.004 | -0.105 | 0.049 | -0.192 | 0.104 | -0.003 | 0.136 | -0.114 | 0.088 | 0.193 | 0.06 | -0.047 | -0.025 | 0.201* | 0.218* |
| OCIR | 0.134 | -0.053 | -0.14 | -0.097 | 0.007 | -0.045 | -0.149 | -0.179 | 0.096 | -0.037 | -0.056 | 0.045 | -0.133 | 0.159 | 0.026 |
| washing | 0.073 | -0.252* | -0.028 | -0.115 | 0.029 | -0.066 | -0.128 | -0.085 | 0.051 | 0.035 | -0.165 | -0.029 | -0.102 | 0.182 | 0.159 |
| obsessing | 0.082 | 0.061 | -0.095 | 0.033 | -0.107 | -0.074 | -0.074 | -0.01 | 0.186 | 0.015 | -0.007 | 0.057 | -0.117 | -0.118 | -0.093 |
| hoarding | 0.175 | 0.085 | -0.096 | -0.008 | 0.006 | 0.007 | 0.077 | -0.096 | 0.019 | 0.13 | -0.07 | -0.094 | 0.042 | 0.188 | -0.041 |
| ordering | 0.113 | -0.03 | -0.081 | -0.003 | -0.052 | 0.046 | -0.145 | -0.185 | -0.094 | 0.054 | 0.047 | 0.003 | -0.222* | 0.182 | 0.035 |
| checking | 0.104 | 0.049 | -0.119 | -0.108 | 0.057 | 0.02 | -0.12 | -0.155 | 0.127 | -0.220* | -0.014 | 0.203* | -0.058 | 0.153 | -0.055 |
| mental neutralizing | 0.05 | -0.034 | -0.126 | -0.182 | 0.08 | -0.091 | -0.147 | -0.101 | 0.039 | -0.095 | 0.025 | 0.016 | -0.059 | 0.006 | 0.096 |
| BDI | 0.097 | 0.032 | 0.048 | -0.031 | -0.104 | -0.111 | -0.04 | -0.023 | 0.05 | 0.064 | 0.07 | 0.114 | -0.046 | 0.042 | 0.185 |
| BAI | 0.049 | 0.201* | 0.001 | -0.059 | 0.093 | -0.197 | -0.011 | -0.141 | 0.077 | -0.03 | 0.003 | -0.047 | -0.056 | 0.144 | 0.071 |
| SDS | -0.249* | -0.113 | 0.092 | -0.065 | 0.109 | -0.146 | -0.054 | -0.098 | -0.003 | 0.194 | -0.049 | 0.189 | -0.027 | 0.028 | 0.024 |
| * p<0.05 |  |  |  |  |  |  |  |  |  |  |  |  |  |  |  |

Table S9.Results of Structural-Functional Coupling in 400 Brain Regions

| Colum | Group_T_Statistic | Raw_P_Value |
| --- | --- | --- |
| 1 L_V1 | 0.4345 | 0.6645 |
| 2 L_MST | -1.7550 | 0.0810 |
| 3 L_V6 | 1.2063 | 0.2293 |
| 4 L_V2 | 0.0716 | 0.9430 |
| 5 L_V3 | 1.1392 | 0.2562 |
| 6 L_V4 | 0.1784 | 0.8586 |
| 7 L_V8 | -0.3978 | 0.6912 |
| 8 L_4 | 1.0649 | 0.2884 |
| 9 L_3b | 0.9652 | 0.3358 |
| 10 L_FEF | 0.4956 | 0.6208 |
| 11 L_PEF | -1.1325 | 0.2590 |
| 12 L_55b | 1.4553 | 0.1474 |
| 13 L_V3A | 1.6078 | 0.1097 |
| 14 L_RSC | 0.1548 | 0.8772 |
| 15 L_POS2 | -0.5187 | 0.6047 |
| 16 L_V7 | 2.4286 | 0.0162 |
| 17 L_IPS1 | 0.8271 | 0.4093 |
| 18 L_FFC | 0.5397 | 0.5901 |
| 19 L_V3B | 1.5566 | 0.1214 |
| 20 L_LO1 | -0.0355 | 0.9717 |
| 21 L_LO2 | 1.5046 | 0.1342 |
| 22 L_PIT | -2.3501 | 0.0199 |
| 23 L_MT | -0.4264 | 0.6703 |
| 24 L_A1 | 1.4471 | 0.1497 |
| 25 L_PSL | 0.7984 | 0.4257 |
| 26 L_SFL | -0.0984 | 0.9217 |
| 27 L_PCV | 1.0776 | 0.2827 |
| 28 L_STV | 2.3705 | 0.0189 |
| 29 L_7Pm | 0.7816 | 0.4355 |
| 30 L_7m | 1.9224 | 0.0562 |
| 31 L_POS1 | 0.1994 | 0.8422 |
| 32 L_23d | 0.5398 | 0.5900 |
| 33 L_v23ab | 1.5832 | 0.1152 |
| 34 L_d23ab | 1.9734 | 0.0500 |
| 35 L_31pv | 0.8999 | 0.3694 |
| 36 L_5m | -1.0317 | 0.3036 |
| 37 L_5mv | -1.6787 | 0.0950 |
| 38 L_23c | 1.1123 | 0.2675 |
| 39 L_5L | 1.1138 | 0.2669 |
| 40 L_24dd | 2.3557 | 0.0196 |
| 41 L_24dv | 0.2263 | 0.8212 |
| 42 L_7AL | 2.0880 | 0.0382 |
| 43 L_SCEF | -1.7129 | 0.0885 |
| 44 L_6ma | 0.1444 | 0.8854 |
| 45 L_7Am | -1.1295 | 0.2602 |
| 46 L_7PL | 0.6292 | 0.5300 |
| 47 L_7PC | 0.1571 | 0.8754 |
| 48 L_LIPv | 1.2235 | 0.2228 |
| 49 L_VIP | 0.0326 | 0.9740 |
| 50 L_MIP | -0.9569 | 0.3400 |
| 51 L_1 | 2.3519 | 0.0198 |
| 52 L_2 | 1.2704 | 0.2056 |
| 53 L_3a | 1.4603 | 0.1460 |
| 54 L_6d | 1.5844 | 0.1149 |
| 55 L_6mp | 0.3752 | 0.7080 |
| 56 L_6v | -0.7369 | 0.4622 |
| 57 L_p24pr | 0.7574 | 0.4498 |
| 58 L_33pr | 0.7548 | 0.4514 |
| 59 L_a24pr | -0.6247 | 0.5330 |
| 60 L_p32pr | -0.0239 | 0.9809 |
| 61 L_a24 | 1.1156 | 0.2661 |
| 62 L_d32 | 0.4351 | 0.6640 |
| 63 L_8BM | 1.3259 | 0.1866 |
| 64 L_p32 | 2.3457 | 0.0201 |
| 65 L_10r | 0.8627 | 0.3895 |
| 66 L_47m | 0.9172 | 0.3603 |
| 67 L_8Av | 0.4331 | 0.6654 |
| 68 L_8Ad | 1.5487 | 0.1233 |
| 69 L_9m | 0.2402 | 0.8105 |
| 70 L_8BL | -0.2851 | 0.7759 |
| 71 L_9p | 2.3042 | 0.0224 |
| 72 L_10d | 2.0390 | 0.0430 |
| 73 L_8C | 0.5759 | 0.5654 |
| 74 L_44 | 2.5655 | 0.0111 |
| 75 L_45 | 1.4493 | 0.1490 |
| 76 L_47l | -0.2411 | 0.8098 |
| 77 L_a47r | 1.0759 | 0.2834 |
| 78 L_6r | 1.1972 | 0.2329 |
| 79 L_IFJa | -0.3655 | 0.7152 |
| 80 L_IFJp | -0.1205 | 0.9042 |
| 81 L_IFSp | -0.7659 | 0.4448 |
| 82 L_IFSa | 0.1930 | 0.8472 |
| 83 L_p9-46v | 1.4554 | 0.1474 |
| 84 L_46 | 0.7522 | 0.4529 |
| 85 L_a9-46v | 1.9123 | 0.0575 |
| 86 L_9-46d | 2.9297 | 0.0038 |
| 87 L_9a | 2.2891 | 0.0233 |
| 88 L_10v | 0.9197 | 0.3590 |
| 89 L_a10p | 2.4608 | 0.0148 |
| 90 L_10pp | -0.5826 | 0.5609 |
| 91 L_11l | -0.4984 | 0.6188 |
| 92 L_13l | -0.1000 | 0.9204 |
| 93 L_OFC | 0.3463 | 0.7295 |
| 94 L_47s | -0.2792 | 0.7804 |
| 95 L_LIPd | 0.0205 | 0.9837 |
| 96 L_6a | -0.1425 | 0.8869 |
| 97 L_i6-8 | -0.5105 | 0.6104 |
| 98 L_s6-8 | 0.2485 | 0.8040 |
| 99 L_43 | 2.2263 | 0.0273 |
| 100 L_OP4 | 0.3509 | 0.7260 |
| 101 L_OP1 | 0.4224 | 0.6732 |
| 102 L_OP2-3 | -1.1312 | 0.2595 |
| 103 L_52 | -0.4111 | 0.6815 |
| 104 L_RI | 1.4933 | 0.1372 |
| 105 L_PFcm | 0.3800 | 0.7044 |
| 106 L_PoI2 | 1.0541 | 0.2933 |
| 107 L_TA2 | 1.4341 | 0.1533 |
| 108 L_FOP4 | 1.7732 | 0.0779 |
| 109 L_MI | 1.4042 | 0.1620 |
| 110 L_Pir | 1.6910 | 0.0926 |
| 111 L_AVI | 1.7161 | 0.0879 |
| 112 L_AAIC | 0.1399 | 0.8889 |
| 113 L_FOP1 | 1.7204 | 0.0871 |
| 114 L_FOP3 | 0.7136 | 0.4764 |
| 115 L_FOP2 | 2.1088 | 0.0364 |
| 116 L_PFt | 0.8590 | 0.3915 |
| 117 L_AIP | 1.1869 | 0.2369 |
| 118 L_EC | 1.9622 | 0.0513 |
| 119 L_PreS | -0.1366 | 0.8915 |
| 120 L_H | 0.8341 | 0.4054 |
| 121 L_ProS | 0.5630 | 0.5741 |
| 122 L_PeEc | -0.7216 | 0.4715 |
| 123 L_STGa | 0.8248 | 0.4106 |
| 124 L_PBelt | 0.9813 | 0.3278 |
| 125 L_A5 | 2.0195 | 0.0450 |
| 126 L_PHA1 | 1.1977 | 0.2326 |
| 127 L_PHA3 | 1.3050 | 0.1936 |
| 128 L_STSda | 1.9872 | 0.0485 |
| 129 L_STSdp | 0.4072 | 0.6844 |
| 130 L_STSvp | 0.5912 | 0.5552 |
| 131 L_TGd | 0.5628 | 0.5743 |
| 132 L_TE1a | -0.5463 | 0.5856 |
| 133 L_TE1p | -1.1880 | 0.2364 |
| 134 L_TE2a | 0.6186 | 0.5370 |
| 135 L_TF | -0.4135 | 0.6798 |
| 136 L_TE2p | 1.6058 | 0.1101 |
| 137 L_PHT | 0.7620 | 0.4471 |
| 138 L_PH | -0.3211 | 0.7485 |
| 139 L_TPOJ1 | 0.9499 | 0.3434 |
| 140 L_TPOJ2 | 1.0646 | 0.2885 |
| 141 L_TPOJ3 | 1.1444 | 0.2540 |
| 142 L_DVT | 2.1125 | 0.0361 |
| 143 L_PGp | 1.8557 | 0.0652 |
| 144 L_IP2 | 1.6596 | 0.0988 |
| 145 L_IP1 | -1.5946 | 0.1126 |
| 146 L_IP0 | 0.6608 | 0.5096 |
| 147 L_PFop | 1.0006 | 0.3184 |
| 148 L_PF | 3.3428 | 0.0010 |
| 149 L_PFm | 0.0949 | 0.9245 |
| 150 L_PGi | 1.3539 | 0.1775 |
| 151 L_PGs | -0.4483 | 0.6545 |
| 152 L_V6A | -0.9822 | 0.3273 |
| 153 L_VMV1 | 0.8044 | 0.4222 |
| 154 L_VMV3 | -1.1405 | 0.2556 |
| 155 L_PHA2 | 2.6263 | 0.0094 |
| 156 L_V4t | 0.0449 | 0.9643 |
| 157 L_FST | 0.1414 | 0.8877 |
| 158 L_V3CD | 0.4710 | 0.6382 |
| 159 L_LO3 | -0.4668 | 0.6412 |
| 160 L_VMV2 | -0.5760 | 0.5654 |
| 161 L_31pd | 0.2862 | 0.7750 |
| 162 L_31a | 0.1217 | 0.9033 |
| 163 L_VVC | 1.6231 | 0.1064 |
| 164 L_25 | 0.1329 | 0.8945 |
| 165 L_s32 | 1.3899 | 0.1663 |
| 166 L_pOFC | -1.1666 | 0.2450 |
| 167 L_PoI1 | -2.2373 | 0.0265 |
| 168 L_Ig | 0.2690 | 0.7883 |
| 169 L_FOP5 | 2.9842 | 0.0033 |
| 170 L_p10p | 1.6424 | 0.1023 |
| 171 L_p47r | 1.7351 | 0.0845 |
| 172 L_TGv | 0.1372 | 0.8910 |
| 173 L_MBelt | 2.1037 | 0.0368 |
| 174 L_LBelt | 0.6404 | 0.5228 |
| 175 L_A4 | -0.6182 | 0.5373 |
| 176 L_STSva | 1.3922 | 0.1656 |
| 177 L_TE1m | -1.3737 | 0.1713 |
| 178 L_PI | 0.8894 | 0.3750 |
| 179 L_a32pr | 0.4222 | 0.6734 |
| 180 L_p24 | 1.8499 | 0.0660 |
| 181 R_V1 | 1.0292 | 0.3048 |
| 182 R_MST | 1.5807 | 0.1158 |
| 183 R_V6 | 0.0981 | 0.9220 |
| 184 R_V2 | 2.3342 | 0.0207 |
| 185 R_V3 | 2.5982 | 0.0102 |
| 186 R_V4 | -0.4947 | 0.6214 |
| 187 R_V8 | 0.4566 | 0.6485 |
| 188 R_4 | -0.6929 | 0.4893 |
| 189 R_3b | 0.9056 | 0.3664 |
| 190 R_FEF | 0.7491 | 0.4548 |
| 191 R_PEF | -1.3151 | 0.1902 |
| 192 R_55b | -1.5905 | 0.1135 |
| 193 R_V3A | 0.8061 | 0.4213 |
| 194 R_RSC | 1.3995 | 0.1634 |
| 195 R_POS2 | -0.1354 | 0.8925 |
| 196 R_V7 | 1.0173 | 0.3104 |
| 197 R_IPS1 | 3.0958 | 0.0023 |
| 198 R_FFC | 0.6537 | 0.5142 |
| 199 R_V3B | -0.2952 | 0.7682 |
| 200 R_LO1 | 1.3111 | 0.1915 |
| 201 R_LO2 | 0.0280 | 0.9777 |
| 202 R_PIT | -0.6077 | 0.5442 |
| 203 R_MT | 2.0489 | 0.0420 |
| 204 R_A1 | 1.3406 | 0.1818 |
| 205 R_PSL | 1.1754 | 0.2414 |
| 206 R_SFL | 1.8183 | 0.0707 |
| 207 R_PCV | 1.9359 | 0.0545 |
| 208 R_STV | 1.8628 | 0.0642 |
| 209 R_7Pm | 0.0004 | 0.9997 |
| 210 R_7m | 2.4307 | 0.0161 |
| 211 R_POS1 | 0.7661 | 0.4447 |
| 212 R_23d | -1.9390 | 0.0541 |
| 213 R_v23ab | 0.8241 | 0.4110 |
| 214 R_d23ab | 1.3505 | 0.1786 |
| 215 R_31pv | 0.1794 | 0.8578 |
| 216 R_5m | -1.8831 | 0.0613 |
| 217 R_5mv | -0.1652 | 0.8690 |
| 218 R_23c | 3.6233 | 0.0004 |
| 219 R_5L | 0.7378 | 0.4616 |
| 220 R_24dd | 2.7144 | 0.0073 |
| 221 R_24dv | 0.9424 | 0.3473 |
| 222 R_7AL | 0.4553 | 0.6495 |
| 223 R_SCEF | -0.3386 | 0.7353 |
| 224 R_6ma | -0.4726 | 0.6371 |
| 225 R_7Am | -0.3210 | 0.7486 |
| 226 R_7PL | 0.3206 | 0.7489 |
| 227 R_7PC | 1.5976 | 0.1119 |
| 228 R_LIPv | 0.5279 | 0.5982 |
| 229 R_VIP | 0.7779 | 0.4377 |
| 230 R_MIP | 0.3359 | 0.7373 |
| 231 R_1 | 0.7330 | 0.4645 |
| 232 R_2 | 1.4801 | 0.1406 |
| 233 R_3a | 0.2450 | 0.8067 |
| 234 R_6d | 0.3353 | 0.7378 |
| 235 R_6mp | 1.5440 | 0.1244 |
| 236 R_6v | -0.3648 | 0.7157 |
| 237 R_p24pr | -1.1105 | 0.2683 |
| 238 R_33pr | 0.9843 | 0.3263 |
| 239 R_a24pr | -3.4604 | 0.0007 |
| 240 R_p32pr | -0.7232 | 0.4705 |
| 241 R_a24 | -0.3829 | 0.7023 |
| 242 R_d32 | 1.0470 | 0.2965 |
| 243 R_8BM | -0.0759 | 0.9396 |
| 244 R_p32 | 0.0323 | 0.9743 |
| 245 R_10r | 0.7557 | 0.4509 |
| 246 R_47m | 0.0913 | 0.9274 |
| 247 R_8Av | -0.7463 | 0.4565 |
| 248 R_8Ad | 1.9212 | 0.0563 |
| 249 R_9m | 0.8692 | 0.3860 |
| 250 R_8BL | 1.2724 | 0.2049 |
| 251 R_9p | 0.9723 | 0.3322 |
| 252 R_10d | 1.0808 | 0.2813 |
| 253 R_8C | 0.6212 | 0.5353 |
| 254 R_44 | -1.1242 | 0.2625 |
| 255 R_45 | 0.7204 | 0.4722 |
| 256 R_47l | 0.0079 | 0.9937 |
| 257 R_a47r | 2.1140 | 0.0359 |
| 258 R_6r | -0.9283 | 0.3545 |
| 259 R_IFJa | 0.8158 | 0.4157 |
| 260 R_IFJp | 0.4203 | 0.6748 |
| 261 R_IFSp | 1.5053 | 0.1341 |
| 262 R_IFSa | -0.0070 | 0.9944 |
| 263 R_p9-46v | 1.7958 | 0.0742 |
| 264 R_46 | 0.3029 | 0.7624 |
| 265 R_a9-46v | 1.8507 | 0.0659 |
| 266 R_9-46d | -1.0450 | 0.2975 |
| 267 R_9a | 1.5942 | 0.1127 |
| 268 R_10v | -1.2589 | 0.2097 |
| 269 R_a10p | 0.3059 | 0.7601 |
| 270 R_10pp | -0.4355 | 0.6637 |
| 271 R_11l | 0.9766 | 0.3301 |
| 272 R_13l | 1.8324 | 0.0686 |
| 273 R_OFC | -0.5777 | 0.5642 |
| 274 R_47s | -0.1927 | 0.8474 |
| 275 R_LIPd | -0.0138 | 0.9890 |
| 276 R_6a | -1.6118 | 0.1088 |
| 277 R_i6-8 | 2.3558 | 0.0196 |
| 278 R_s6-8 | -1.5233 | 0.1295 |
| 279 R_43 | -0.9844 | 0.3263 |
| 280 R_OP4 | -0.4629 | 0.6440 |
| 281 R_OP1 | 0.9091 | 0.3646 |
| 282 R_OP2-3 | 1.0577 | 0.2916 |
| 283 R_52 | 0.3286 | 0.7429 |
| 284 R_RI | 2.1002 | 0.0371 |
| 285 R_PFcm | -1.3907 | 0.1661 |
| 286 R_PoI2 | 1.4423 | 0.1510 |
| 287 R_TA2 | 1.1059 | 0.2703 |
| 288 R_FOP4 | 1.1819 | 0.2389 |
| 289 R_MI | -0.1739 | 0.8622 |
| 290 R_Pir | -0.6977 | 0.4863 |
| 291 R_AVI | 1.0316 | 0.3037 |
| 292 R_AAIC | -0.8467 | 0.3983 |
| 293 R_FOP1 | 0.3910 | 0.6963 |
| 294 R_FOP3 | 0.8120 | 0.4179 |
| 295 R_FOP2 | 1.6670 | 0.0973 |
| 296 R_PFt | 1.5946 | 0.1126 |
| 297 R_AIP | -1.9281 | 0.0555 |
| 298 R_EC | 1.4749 | 0.1420 |
| 299 R_PreS | -0.6224 | 0.5345 |
| 300 R_H | 0.4283 | 0.6690 |
| 301 R_ProS | 1.9530 | 0.0524 |
| 302 R_PeEc | 1.3602 | 0.1755 |
| 303 R_STGa | -0.4639 | 0.6433 |
| 304 R_PBelt | 0.6418 | 0.5218 |
| 305 R_A5 | 0.3929 | 0.6949 |
| 306 R_PHA1 | 1.2969 | 0.1964 |
| 307 R_PHA3 | 1.5540 | 0.1220 |
| 308 R_STSda | 0.2351 | 0.8144 |
| 309 R_STSdp | 1.3390 | 0.1823 |
| 310 R_STSvp | 1.6930 | 0.0922 |
| 311 R_TGd | 0.3736 | 0.7092 |
| 312 R_TE1a | 0.9247 | 0.3564 |
| 313 R_TE1p | 0.6317 | 0.5284 |
| 314 R_TE2a | 1.8134 | 0.0715 |
| 315 R_TF | 3.3358 | 0.0010 |
| 316 R_TE2p | -0.2492 | 0.8035 |
| 317 R_PHT | -0.4744 | 0.6358 |
| 318 R_PH | 0.5551 | 0.5795 |
| 319 R_TPOJ1 | 2.5420 | 0.0119 |
| 320 R_TPOJ2 | 2.6295 | 0.0093 |
| 321 R_TPOJ3 | -0.8195 | 0.4136 |
| 322 R_DVT | 1.5152 | 0.1315 |
| 323 R_PGp | 0.0488 | 0.9611 |
| 324 R_IP2 | 0.4008 | 0.6891 |
| 325 R_IP1 | -0.5504 | 0.5827 |
| 326 R_IP0 | 0.7081 | 0.4798 |
| 327 R_PFop | 1.2730 | 0.2047 |
| 328 R_PF | -0.2203 | 0.8259 |
| 329 R_PFm | 1.6295 | 0.1050 |
| 330 R_PGi | 1.0930 | 0.2759 |
| 331 R_PGs | 0.7341 | 0.4639 |
| 332 R_V6A | 0.5952 | 0.5525 |
| 333 R_VMV1 | 0.9818 | 0.3275 |
| 334 R_VMV3 | -0.0161 | 0.9871 |
| 335 R_PHA2 | 0.1379 | 0.8905 |
| 336 R_V4t | -1.6567 | 0.0994 |
| 337 R_FST | 0.9032 | 0.3677 |
| 338 R_V3CD | 0.0250 | 0.9801 |
| 339 R_LO3 | -0.9104 | 0.3638 |
| 340 R_VMV2 | -1.3838 | 0.1682 |
| 341 R_31pd | -0.9707 | 0.3330 |
| 342 R_31a | 2.1572 | 0.0324 |
| 343 R_VVC | 0.6499 | 0.5166 |
| 344 R_25 | 1.0737 | 0.2844 |
| 345 R_s32 | -0.3586 | 0.7203 |
| 346 R_pOFC | -0.4692 | 0.6395 |
| 347 R_PoI1 | -0.2953 | 0.7681 |
| 348 R_Ig | 0.5918 | 0.5548 |
| 349 R_FOP5 | -0.4256 | 0.6709 |
| 350 R_p10p | 2.6107 | 0.0098 |
| 351 R_p47r | 0.4841 | 0.6289 |
| 352 R_TGv | -0.0962 | 0.9234 |
| 353 R_MBelt | 0.9275 | 0.3549 |
| 354 R_LBelt | 1.9485 | 0.0530 |
| 355 R_A4 | -1.0436 | 0.2981 |
| 356 R_STSva | 1.5541 | 0.1220 |
| 357 R_TE1m | -0.2402 | 0.8105 |
| 358 R_PI | 0.8132 | 0.4172 |
| 359 R_a32pr | -0.5225 | 0.6020 |
| 360 R_p24 | 0.0364 | 0.9710 |
